# Past, current, and future trends in the prevalence of primary sclerosing cholangitis and inflammatory bowel disease across England (2015–2027): a nationwide, population-based study

**DOI:** 10.1016/j.lanepe.2024.101002

**Published:** 2024-07-10

**Authors:** Hannah Crothers, James Ferguson, Mohammed Nabil Quraishi, Rachel Cooney, Tariq H. Iqbal, Palak J. Trivedi

**Affiliations:** a Research and Development, University Hospitals Birmingham NHS Foundation Trust, Birmingham B15 2TH, UK; b Liver Unit, University Hospitals Birmingham NHS Foundation Trust, Birmingham B15 2TH, UK; c National Institute for Health and Social Care Research (NIHR) Birmingham Biomedical Research Centre (BRC), Centre for Liver and Gastrointestinal Research, University of Birmingham, Birmingham B15 2TT, UK; d Department of Gastroenterology, University Hospitals Birmingham NHS Foundation Trust, Birmingham B15 2TH, UK; e Institute of Immunology and Immunotherapy, University of Birmingham, B15 2TT, UK; f Institute of Applied Health Research, University of Birmingham, B15 2TT, UK

**Keywords:** Autoimmune liver diseases, Cholestasis, Epidemiology, Rare disease

## Abstract

**Background:**

Primary sclerosing cholangitis (PSC) is one of the leading indications for liver transplantation in Europe, and a major risk factor for cancer in inflammatory bowel disease (IBD). However, it is not known how the epidemiology of PSC will change as that of IBD evolves. The aim of this study is to provide nationwide statistics on the past and current prevalence of PSC and IBD across England, and forecast how this is likely to change over time.

**Methods:**

We accessed and analysed a nationwide population-based administrative healthcare registry, which houses prospectively accrued data since April 1st 2001. In so doing, the past and current prevalence of PSC-IBD and IBD alone was determined among 18–60-year-olds in England, alongside average annual percentage change rates (AAPC), between the 1st of January 2015 and 2020. Past and current prevalence data, alongside trends in incidence and event-free survival rates, were then used to forecast future prevalence between 2021 and 2027.

**Findings:**

In 2015, the prevalence of PSC with prior IBD diagnosis was 5.0 per 100,000 population, rising to 5.7 when including those with IBD diagnosed after PSC. In 2020, prevalence increased to 7.6 (8.6 accounting for IBD developing after PSC), yielding an AAPC of 8.8. In 2027, PSC-IBD prevalence is forecast to be 11.7 (95% prediction interval [PI]: 10.8–12.7), and 13.3 when accounting for IBD developing after PSC (AAPC: 6.4; 95% PI: 5.3–7.5). Comparatively, the prevalence of IBD alone rose among 18–60-year-olds from 384.3 in 2015 to 538.7 in 2020 (AAPC 7.0), and forecast to increase to 742.5 by 2027 (95% PI: 736.4–748.0; AAPC: 4.7, 95% PI: 4.6–4.8).

**Interpretation:**

The rate of growth in PSC-IBD is predicted to exceed IBD-alone. Further research is needed to understand changes in disease epidemiology, including aetiological drivers of developing (invariably progressive) liver disease in IBD, and the implications of rising case burden on health care resources.

**Funding:**

This study was supported by an unrestricted grant provided by 10.13039/100005564Gilead Sciences.

**Research in context**

**Evidence before this study**

Primary sclerosing cholangitis (PSC) is a rare and invariably progressive liver condition that predominantly affects people with inflammatory bowel disease (IBD), most often men. We conducted and published a systematic literature review following a search of MEDLINE and EMBASE, from database inception up to June 2020, to identify population-based studies reporting the epidemiology of PSC. Studies were identified from MEDLINE using search terms: (“Cholangitis, Sclerosing”[MeSH] OR “primary sclerosing cholangitis”) AND (“epidemiology” OR “incidence” OR “prevalence” OR “incident” OR “prevalent”). EMBASE was searched using the terms: (‘primary sclerosing cholangitis’/exp OR ‘primary sclerosing cholangitis’ OR ‘primary sclerosing cholangitis’:ab,ti) AND (‘epidemiology’/exp OR ‘epidemiology’ OR ‘incidence’/exp OR ‘incidence’ OR inciden:ab,ti OR ‘prevalence’/exp OR ‘prevalence’ OR prevalen:ab,ti). An increase in regional prevalence of PSC was reported in four studies (three from Europe conducted between 1986–1995, 2000–2008 and 1998–2014); however, none were whole country/nationwide descriptors. Additionally, there were no population-based studies, at regional or national level, comparing temporal changes in PSC prevalence to that of IBD alone, and how this is forecasted to evolve over time. A further, independent systematic review published by another group, conducted by searching MEDLINE and EMBASE (from database inception up to the 10th of August 2023), did not find any additional studies of relevance. Thus, to the best of our knowledge, there are no nationwide, population-based data relating to the prevalence of PSC in England or other countries, how this is predicted to change, or any head-to-head comparative data to IBD alone.

**Added value of this study**

This study shows that the prevalence rates of PSC-IBD in England have risen since 2015 and are forecast to increase over time. Additionally, the growth rate is predicted to outpace that of IBD alone, predominantly affecting 30- to 44-year-olds, and with the rate of growth of PSC in women reaching that seen in men. Moreover, the regions of peak PSC-IBD prevalence in England differ to those of IBD alone. Whilst the prevalence of IBD alone is also predicted to rise over time, the growth rate between 2021 and 2027 is expected to decline, without differences between sexes or IBD type.

**Implications of all the available evidence**

Identifying the aetiological drivers of PSC amongst IBD patients, particularly those that are exposomal and environmental, are critical to furthering our understanding of epidemiology and disease pathogenesis. Moreover, the increased rate of growth of PSC-IBD has implications for healthcare providers, particularly those working in liver transplant units, clinical trialists, and for future health economic modelling.

## Introduction

Primary sclerosing cholangitis (PSC) is the classic hepatobiliary manifestation of inflammatory bowel disease (IBD).^1^, ^2^, ^3^ Phenotypically, disease is characterized by multifocal strictures throughout the biliary tree, with clinical outcomes being dictated by the onset of recurrent biliary infections, cirrhosis, and a predisposition toward bowel and hepatobiliary cancers. Despite advances in IBD therapeutics, liver transplantation remains the only life-extending intervention for patients, with a median transplant-free survival of 14–21 years from diagnosis. Indeed, whilst PSC is a rare disease, it is one of the leading indications for transplantation in the UK, France and the Nordic countries.^4^, ^5^, ^6^, ^7^

However, the horizon for new treatments is encouraging, given innovative clinical trial programmes from the commercial sector, alongside a wealth of academic studies.^8^ Whilst regulatory approval is critical for getting drugs to market, it no longer guarantees market access. With growing emphasis on economic modelling, recommendations for reimbursement pose an additional hurdle for getting new therapies into Europe. Importantly, the premise of any cost analysis is reliant on robust understandings of disease prevalence, how this is forecast to change, and the projected burden of disease on health care services.

The global epidemiology of IBD has been well defined, with contemporary data indicating that Europe has entered a phase of compounding prevalence.^9^ However, with regards PSC-IBD specifically, the incidence appears to be rising, despite stabilisation in the rate of new IBD presentations.^1^ Regardless, the scale of unmet need is not well defined, and it is not known how the prevalence of PSC-IBD is changing as that of IBD evolves. This is particularly relevant given the high rates of liver transplantation and PSC-related mortality,^1^^,^^2^ in an era devoid of definitive medical therapy.

The overarching goal of this study is to provide nationwide statistics on PSC and IBD epidemiology in England using routinely collected administrative healthcare data. In so doing, we sought to better understand how the prevalence of PSC-IBD differs to that of IBD alone according to geography and patient demographics, and how the epidemiology of these diseases is forecast to change over time.

## Methods

Our study comprised three principal aims: (1) to quantify the past and current prevalence of PSC-IBD and IBD alone between the 1st of January 2015 to the 1st of January 2020, (2) construct a well-fitting forecast model, capable of predicting current disease prevalence (1st of January 2018 to the first of January 2020) using epidemiological data from the years prior, and see how well these predicted rates mirror those actually observed in the same time frame; and (3) extrapolate the latter methodology, to construct forecasting models capable of predicting future disease prevalence (between the 1st of January 2021 to the 1st January 2027).

### Study population and overview of data sources

This was a nationwide population-based study conducted throughout the whole of England, performed via auditing of patient medical records held by the Hospital Episode Statistics (HES) registry and the Office of National Statistics (ONS). Notably, HES data encompasses all National Health Service (NHS) Clinical Commissioning Groups in England, detailing every hospital attendance, admission, and investigative and therapeutic procedure undertaken in England. Fuller details pertaining to the scale of data capture within HES are given elsewhere,^1^^,^^10^ and summarized in Supplementary File 1.

Primary sclerosing cholangitis is defined by either:

(a) radiological features of sclerosing cholangitis (e.g., biliary irregularities, stenoses or stricturing) on magnetic resonance imaging (MRI) cholangiopancreatography or endoscopic retrograde cholangiopancreatography, or;
(b) biliary disease evident on a liver biopsy, which bears compatible histological features (e.g., fibrosing cholangiopathy, a biliary pattern of interface activity, bile duct loss, or changes relating to chronic cholestasis); in the absence of other causes of biliary and/or other known chronic liver or biliary disease.
  Importantly for our study, only patients with PSC and concomitant IBD were studied.^11^

To gain insight into accessibility and availability of imaging, as relates to MRI scans, we accessed (a) the National Imaging Data Collection in England, which details the number of MRI scanners by geographical region; and (b) the Diagnostic Imaging Dataset (DID),^12^^,^^13^ which is a resource that provides summative data relating to the number of scans performed per year by region standardised by population density. Of note, the latter provides data by individual healthcare board across England, whereas the former provides data in line with broader geographical regions.

### Determining past and current prevalence

**Fig. 1:**
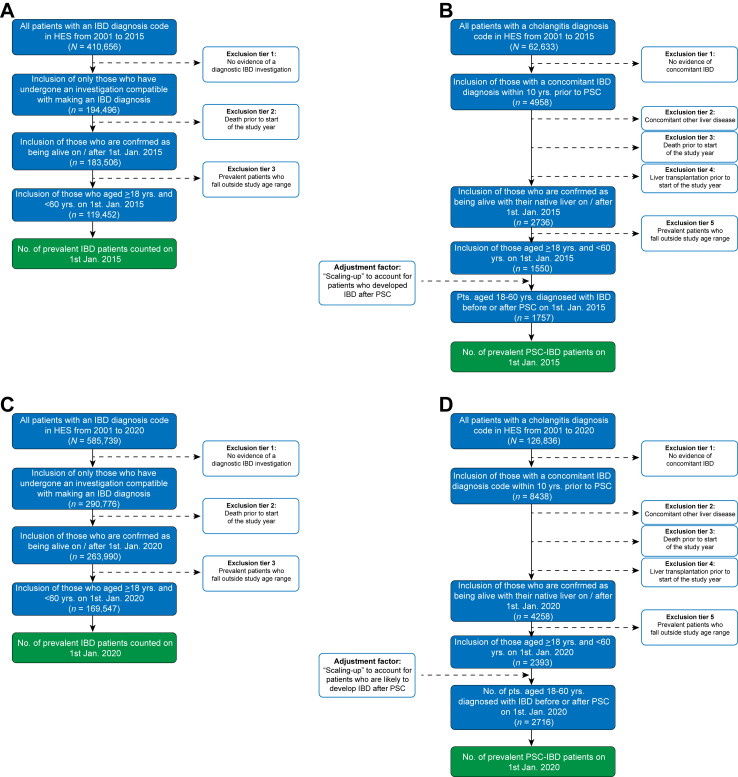
**Identification of past and current prevalent cases of IBD, with and without PSC. (A)** The method for identifying prevalent cases of IBD on the 1st of January 2015 is shown. All patients with a diagnosis of IBD from 2001 onward were identified first, following which any individual who had not undergone an investigation compatible with making an IBD diagnosis were excluded. Thereafter, current prevalence was estimated by only including patients who were still alive at a particular study date (e.g., for prevalence on January 1st 2015, any individual who had died prior to this date were no longer counted). Lastly, patients who were below 18 years or above 60 years of age were removed from downstream analysis. **(B)** The method for identifying prevalent cases of PSC-IBD on the 1st of January 2015 is shown. First, all patients with diagnosis of sclerosing cholangitis were identified, and those without evidence of prior IBD diagnosis excluded as were individuals with a concomitant diagnosis of another liver disease code. Thereafter, current prevalence was estimated by only including patients who were still alive at the given study date. Patients who were below 18 years or above 60 years of age were then removed from downstream analysis. Lastly, patients that were alive and whose ages fell within the pre-specified age, who developed IBD up to five years after PSC diagnosis were then counted and included in final analysis of PSC-IBD prevalence. The same process outlined in panels **(A)** and **(B)** was performed for subsequent years in the principal study period (2016–2020), for IBD with and without PSC. Accordingly, the method for identifying prevalent cases of IBD on the 1st of January 2020, the end of the principal study period, is shown in **(C)**, and for prevalent cases of PSC-IBD on the 1st of January 2020 in **(D)**. Abbreviations: IBD, inflammatory bowel disease; PSC, primary sclerosing cholangitis.

An overview of study timelines, including for data extraction, the principal study period, and forecasting dates relating to future disease prevalence, are summarised in Supplementary File 1 and Supplementary Table S1. Briefly, the principal study period in which annual disease prevalence was determined began on the 1st of January 2015 (Fig. 1A and B). Annual disease prevalence was then quantified on the 1st of January of each consecutive year (2016, 2017, 2018, and 2019) up to and including the 1st of January 2020 (Fig. 1C and D). However, we interrogated the HES registry from the 1st of April 2001 (the first available date of national inpatient data) and the 1st of April 2006 (the first available date of national outpatient data) up until the 31st of December 2019. This is because patients with IBD were only counted in prevalence estimates if there was evidence they had undergone an investigation or practical procedure in the past that was able to make a diagnosis (such as compatible imaging, a colonoscopy, or flexible sigmoidoscopy). Similarly, patients with PSC were only counted in prevalence estimates if a sclerosing cholangitis code was attributed, and they had undergone an investigation compatible with making a PSC diagnosis at some point (such as a previous magnetic resonance cholangiogram, endoscopic retrograde cholangiogram, or liver biopsy). Additionally, patients with concomitant coding for another chronic liver disease at any point were also excluded, as previously described (Supplementary Table S2).^1^ Exclusion criteria with regards other concomitant liver diseases encompassed coding for any of the following conditions: primary biliary cholangitis, alcohol related liver disease, metabolic dysfunction associated steatotic liver disease, vascular liver disorders, chronic viral hepatitis B or C, haemochromatosis, alpha 1 antitrypsin deficiency or Wilson disease.

As per previous descriptors of PSC using HES data, only patients with PSC and concomitant IBD were studied, given that coding for PSC without IBD is less accurate.^1^ In so doing, patients with PSC were only counted in prevalence estimates between 2015 and 2020, if there was evidence of an IBD diagnosis recorded within 10 years before PSC diagnosis. Thereafter, we quantified the number of patients who developed IBD up to five years after PSC diagnosis for each geographic region in England in the year 2015, to provide a corrected estimate of PSC-IBD prevalence nationally. The same correction factor was used to estimate the prevalence of patients inclusive of those who developed IBD within five years after PSC diagnosis for subsequent study years and when applying forecasting models. The five-year prospective time frame was chosen because among patients who develop IBD after PSC, the majority do so within this interval. Censoring of prevalent cases was performed in the event of death or liver transplantation.

Additionally, the age range for study was prespecified as >/=18 years and </= 60 years’ old. An arbitrary upper age band was chosen as clinical coding (with regards IBD and PSC) is less accurate in older age.^14^^,^^15^ Notably, in a previous study looking at admissions related to IBD, coding was found to be 94.3% accurate; but when split by age only 3% of patients aged between 18 and 60 were inaccurately coded compared with 13% of those aged over 60 years. A similar analysis carried out at a neighbouring hospital trust found a miscoding rate in patients aged 18–60 years of 5.0%, but this increased to 25% in patients aged >60 years.^16^ Moreover, people of younger presenting age are those who most often experience a PSC-related clinical event and in greatest need of new therapies.^1^

### Forecasting disease prevalence

Next, we set out to forecast disease prevalence; initially for a period where the actual prevalence had already been quantified (1st January 2018 to 2020), and then for future years, where prevalence rates are unknown (1st January 2021 to 2027). The statistical methods for forecasting prevalence were adapted from those developed by Keogh et al., for the rare disease cystic fibrosis.^17^

**Fig. 2:**
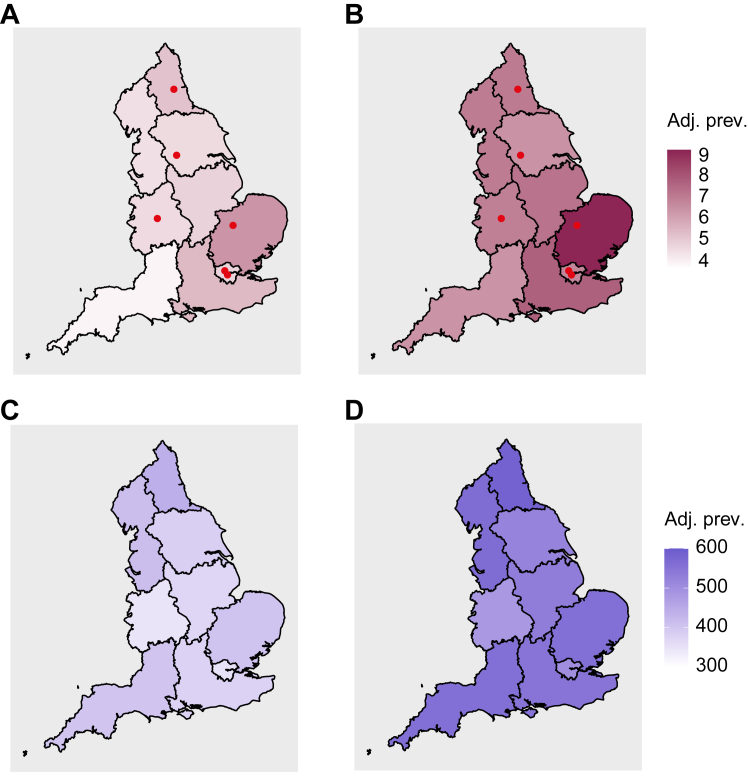
**The prevalence of PSC-IBD and IBD in England.** The sex- and age-adjusted prevalence per 100,000 population is presented for PSC-IBD in 2015 **(A)** and PSC-IBD in 2020 **(B)**. The sex- and age-adjusted prevalence per 100,000 population is presented for IBD alone in 2015 **(C)** and for IBD alone in 2020 **(D)**. Choropleth maps indicate the location of patients at the time of study, according to geographic region. Hierarchical colour coding indicates the regions of greatest (dark) to lowest prevalence (light). Red circles indicate the location of tertiary specialist liver units.

As such, the methods used for forecasting disease prevalence per year require the following information as input (Supplementary Fig. S1):

(1) The number of prevalent cases at the start of the forecasting period (Fig. 1);
(2) An estimate of how many incident cases of disease occur in each year prior, and an estimation of how many new incident cases will account for prevalent cases in later years;
(3) The age-specific probability of a patient meeting a clinical endpoint event in a given year (liver transplantation or death); i.e., an event that would remove them being classified as a ‘prevalent case of PSC’; and
(4) Projections of how the overall population of England is likely to change over time (obtained from the Office of National Statistics [ONS]-based population projections for England).

First, several models of forecasting prevalence were created based on age, sex and IBD type, using prevalence data from 2015 to 2017, alongside incidence and event free survival rates from years prior (Supplementary File 1 and Table S3). The forecasted prevalence from each model was compared to the actual observed prevalence between 2018 and 2020, which had already been quantified in the prior step. The method associated with the best fitting model (in which predicted prevalence most closely mirrored actual observed prevalence between 2018 and 2020) was then extrapolated upon to create models capable of forecasting future disease prevalence between 2021 and 2027. Other model combinations for forecasting disease prevalence are also presented as Supplementary Material where indicated, and used as sensitivity analysis.

Age-specific prevalence forecasts were calculated iteratively. For example, the number of individuals of a particular age with PSC-IBD at the start of the next year was calculated as the number of individuals one year younger with PSC-IBD at the start of the current year, multiplied by their probability of surviving transplant-free for that year, plus the age-specific number of incident cases in the current year that survive transplant-free until the end of the year (Supplementary File 1). This iteration was run seven times to project prevalence estimates seven years forward from the start date.

95% prediction intervals were constructed by generating 500 random draws of incidence rates and survival probabilities and using each set of incidence rates and survival probabilities to obtain 50 draws of the numbers of incident cases and surviving prevalent cases from binomial distributions. This resulted in a set of 25,000 predictions of prevalence, the 2.5th and 97.5th percentiles of which define an interval within which the actual prevalence is expected to lie with probability 0.95.

Average annual percent change (AAPC) across multiple years was calculated as the mean of the annual percent changes for each pair of consecutive years. For example, for the period 2020–2027 there are 7 pairs of consecutive years and so:

$$\text{AAPC}\;=\;\frac{\sum_{y=2021}^{2027}\frac{p_{y}}{p_{y-1}}\;-1}{7}\;\times \;100$$

where $p_{y}$ is the prevalence in year y.

To calculate prediction intervals for the AAPC, this calculation was done for each of the 25,000 predictions of prevalence and the relevant percentiles of the resulting distribution of AAPC values were taken.

### Ethics and regulatory approval

Institutional regulatory board approval was obtained before study initiation and database/chart review (UHB CARMS-16209).

### Role of the funding source

This study was supported by an unrestricted grant provided by Gilead Sciences, awarded to the University of Birmingham (UK). The grant funder had no role in study design, data collection, data extraction, data interpretation, data analysis, manuscript preparation, or in the decision to submit the paper for publication.

## Results

### National and regional estimates of disease prevalence

The 1st of January 2015 was the first date that we determined disease prevalence nationally (Fig. 1). As such, the point prevalence of PSC-IBD in England was estimated at 5.0 per 100,000 population, increasing by 13.5% to 5.7 per 100,000 when accounting for patients who developed IBD after PSC (Supplementary Fig. S2).

The region with peak PSC-IBD prevalence was the East of England (6.4 per 100,000), with the lowest being the Southwest (3.8 per 100,000) (Fig. 2). Men represented the principal affected demographic (prevalence 6.8 per 100,000), alongside those with concomitant UC (Table 1) (Supplementary Table S4).

**Table 1:** Disease prevalence from 2015 to 2020.

A. PSC-IBD														
Group	2015		2016		2017		2018		2019		2020		Overall % change (2015–2020)	
	Adj.^a^	Corr.^b^	Adj.^a^	Corr.^b^	Adj.^a^	Corr.^b^	Adj.^a^	Corr.^b^	Adj.^a^	Corr.^b^	Adj.^a^	Corr.^b^	Adj.^a^	Corr.^b^
All	5.0	5.7	5.5	6.2	6.1	6.9	6.6	7.5	7.1	8.0	7.6	8.6	52%	51%
Sex														
Male	6.8	7.7	7.4	8.4	8.0	9.1	8.7	9.9	9.3	10.5	9.8	11.1	44%	44%
Female	3.2	3.6	3.6	4.1	4.1	4.6	4.5	5.1	4.9	5.6	5.4	6.1	69%	69%
IBD type														
CD	1.1	1.2	1.1	1.2	1.3	1.5	1.4	1.6	1.6	1.8	1.7	1.9	55%	58%
UC	3.9	4.4	4.4	5.0	4.8	5.4	5.1	5.8	5.5	6.2	5.9	6.7	51%	52%
Age group														
18–29 y	5.0	5.7	5.7	6.5	6.2	7.0	6.6	7.5	7.1	8.0	7.6	8.6	52%	51%
30–44 y	4.4	5.0	5.1	5.8	5.8	6.6	6.4	7.3	7.1	8.0	7.9	9.0	80%	80%
45–60 y	5.5	6.2	5.7	6.5	6.2	7.0	6.6	7.5	7.1	8.0	7.3	8.3	33%	34%

B. IBD alone							
	Adj.^a^	Adj.^a^	Adj.^a^	Adj.^a^	Adj.^a^	Adj.^a^	Overall % change (2015–2020)
All	384.3	414.4	445.7	477.0	507.8	538.7	40%
Sex							
Male	374.3	403.3	434.9	465.9	496.3	527.0	41%
Female	394.3	425.4	456.6	488.3	519.4	550.4	40%
IBD type							
CD	146.6	158.1	169.7	181.3	193.0	204.9	40%
UC	237.7	256.3	276.1	295.7	314.9	333.8	40%
Age group							
18–29 y	264.5	285.0	304.0	323.3	338.1	352.8	33%
30–44 y	403.1	436.8	470.3	503.6	538.3	574.4	42%
45–60 y	456.6	489.3	526.8	563.7	601.2	637.7	40%

Abbreviations: CD, Crohn’s disease; IBD, inflammatory bowel disease; PSC, primary sclerosing cholangitis; UC, ulcerative colitis.

a Adjusted prevalence in 18–60 year-olds on 1st January, per 100,000 population.

b Corrected prevalence accounting for patients with PSC who develop IBD up to five years after PSC.

We found that only a few people with PSC-IBD had a diagnosis of IBD unclassified (or indeterminate colitis [IC]), of whom a large proportion had their diagnosis revised to ulcerative colitis (UC) within the following three years. Thus, the PSC-IC group and PSC-UC group were grouped together moving forward. Overall, PSC-UC prevalence was found to be approximately 4-fold greater than PSC with Crohn’s disease (CD) (4.4 vs 1.2 per 100,000).

Comparatively, the estimated prevalence of IBD alone in 2015 among 18–60-year-olds was 384.3 per 100,000 population, peaking in the Northeast (440.8) and being lowest in the West Midlands (346.3). The predominant phenotype of IBD reported was also UC, albeit with similar prevalence rates observed between sexes (Table 1) (Supplementary Table S5).

### The growth rate of PSC-IBD in England compared to IBD alone

In 2020, the prevalence of PSC-IBD rose to 7.6 per 100,000 (increasing to 8.6 when accounting for patients developing IBD after PSC) and of IBD alone to 538.7 per 100,000, respectively. The regions of peak PSC-IBD still differed to those of IBD-alone (Fig. 2), as did the predominant demographic subgroups for each condition (Table 1).

Over time, the total number of MRI scans performed in England was similar in 2015 and 2020 (Supplementary Fig. S3). Moreover, regions of heightened PSC-IBD prevalence did not fall within areas having a greater number of MRI scanners, nor did they localise to healthcare boards with greater population-density standardised MRI rates over time (Supplementary Figs. S3 and S4).

**Fig. 3:**
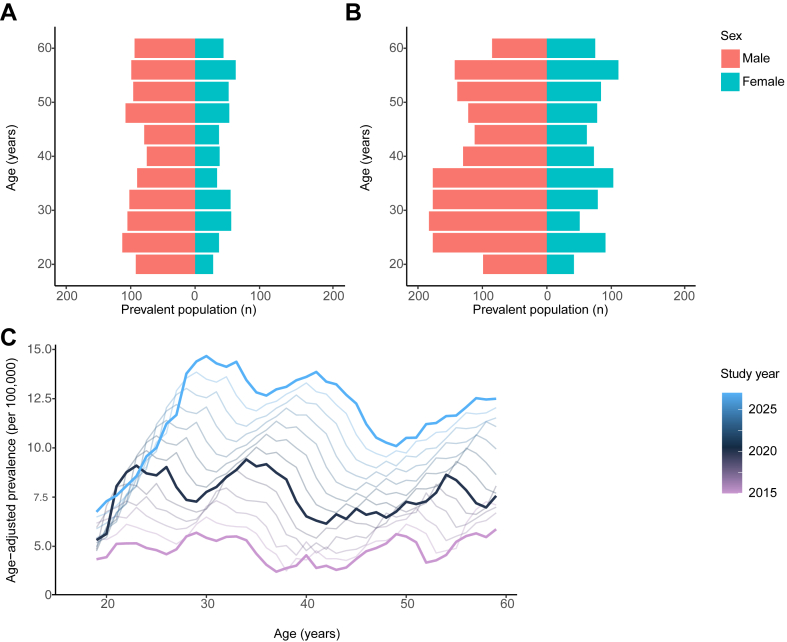
**Prevalence of PSC-IBD in England according to age. (A)** Population pyramids are shown indicating the split of patients with PSC-IBD according to age and sex in the year 2015; and **(B)** in 2020. **(C)** The line graph illustrates changes to the age distribution of PSC-IBD, as relates to the past, current and future forecasted prevalence.

The rate of growth of PSC-IBD, as reflected by the AAPC between 2015 and 2020, was 8.8 (95% CI: 7.1–10.5), compared to 7.0 for IBD alone (95% CI: 6.1–7.9). A larger growth rate in PSC-IBD prevalence was seen among women than men and those with PSC-CD compared to PSC-UC, with peak prevalence occurring in those aged 30–44 years (Table 2 and Fig. 3). Comparatively, the rate of growth for IBD alone was similar between sexes and IBD types, and peak prevalence observed among people aged 45–60 years. Taking the year 2015 as a reference point, the relative risk of IBD patients living with PSC increased to 1.09 (95% CI 1.02, 1.16) in 2020 (Supplementary Table S6).

**Table 2:** Past and forecasted rate of changes in disease prevalence.

A. PSC-IBD				
Group	AAPC 2015–2020	(95% CI)	AAPC 2020–2027	(95% PI)
All	8.8	(7.1, 10.5)	6.4	(5.3, 7.5)
Sex				
Male	7.6	(6.1, 9.1)	6.5	(5.4, 7.7)
Female	11.1	(7.8, 14.4)	6.1	(4.8, 7.4)
IBD type				
CD	10.3	(7.3, 13.3)	6.7	(4.3, 9.3)
UC	8.4	(6.3, 10.4)	6.3	(5.1, 7.5)
Age				
18–29 y	8.8	(5.2, 12.5)	3.5	(2.1, 5.1)
30–44 y	12.3	(9.6, 15.0)	8.0	(6.8, 9.2)
45–60 y	5.9	(3.2, 8.5)	6.5	(5.3, 7.8)
**B. IBD Alone**				

All	7.0	(6.1, 7.9)	4.7	(4.6, 4.8)
Sex				
Male	6.9	(6.0, 8.0)	4.7	(4.5, 4.9)
Female	7.1	(5.9, 7.8)	4.7	(4.5, 4.9)
IBD type				
CD	6.9	(6.1, 7.7)	4.9	(4.7, 5.1)
UC	7.0	(6.0, 7.9)	4.6	(4.4, 4.7)
Age				
18–29 y	5.9	(5.8, 7.5)	1.7	(1.4, 2.0)
30–44 y	7.3	(4.1, 7.8)	5.4	(5.2, 5.5)
45–60 y	6.9	(6.2, 7.7)	5.2	(5.1, 5.3)

Abbreviations: AAPC, average annual percentage change in disease prevalence; CD, Crohn’s disease; IBD, inflammatory bowel disease; 95% CI, 95% confidence interval; 95% PI, 95% prediction interval; PSC, primary sclerosing cholangitis; UC, ulcerative colitis.

### Developing a model to forecast disease prevalence

Next, we combined estimates of trends in disease incidence and survival (Supplementary Figs. S5–S8), alongside disease prevalence rates between 2015 and 2017, and estimated nationwide population projections across England, to develop a model capable of forecasting current disease prevalence (between years 2018 and 2020). For PSC-IBD, the best-fitting model, in which forecast prevalence between years 2018–2020 most closely mirrored actual observed prevalence, contained interaction terms between IBD subtype and each of age, sex and log(year—2000). For IBD the best-fitting model contained interaction terms between IBD type and the three other predictors, as well as between age and log(year—2000) and age and sex (Supplementary Fig. S9 and Table S3). These models were henceforth extrapolated upon, to forecast future disease prevalence moving forward.

### Predicting the future prevalence of PSC-IBD

**Fig. 4:**
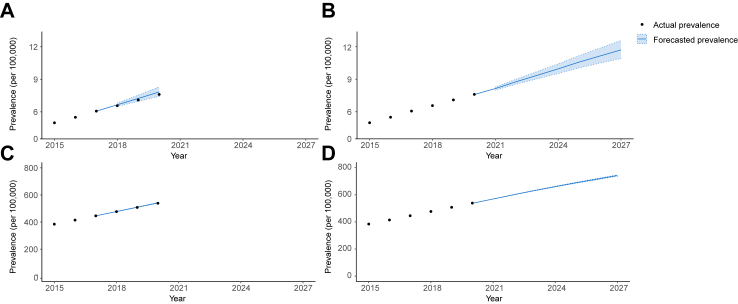
**Actual and forecasted prevalence for PSC-IBD and IBD alone. (A)** The prevalence of PSC-IBD is shown until the 2020. Years 2017–2020 were used as a calibration set, wherein the actual prevalence (black circles) is compared to the estimated prevalence (solid lines) with 95% predicted intervals (dashed lines), forecast from the years prior, alongside estimated incidence and mortality models adjusted for IBD subtype, age category, sex and log(year-2000). Many different models of this type were fitted, reflecting the possible options for interactions between variables. **(B)** The future prevalence of PSC-IBD, forecast to 2027 is shown, using the “adjusted” incidence and mortality models. **(C)** The current and forecasted prevalence of IBD alone is shown, using the “adjusted” incidence and mortality models as described previously. **(D)** The future prevalence of IBD alone, forecast to 2027 is shown using the “adjusted” model as described previously.

**Fig. 5:**
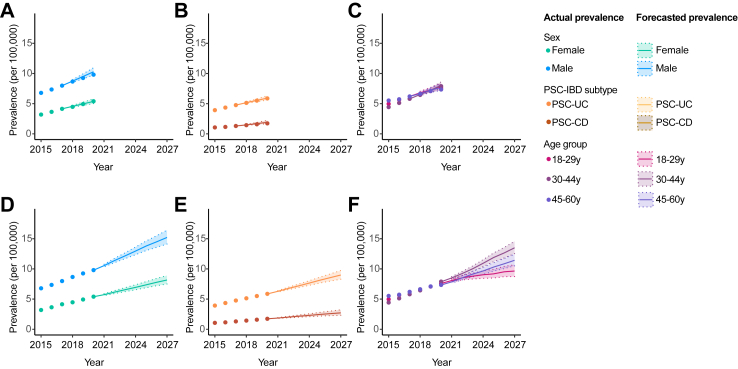
**Actual and forecasted prevalence for PSC-IBD by subgroup. (A–C)** The prevalence of PSC-IBD is shown until the 2020. Years 2017–2020 were used as a calibration set, wherein the actual prevalence (circles) is compared to the estimated prevalence (solid lines) with 95% prediction intervals (dashed lines) forecast from the years prior, stratified by sex **(A)**, IBD subtype (**B)**, and age group **(C)**. In **(D–F)** The future prevalence of PSC-IBD, forecast to 2027 is also shown, stratified by sex **(D),** IBD subtype **(E)**, and age group **(F)**. The future prevalence of IBD alone, forecast to 2027 is shown using the “adjusted” model as described previously.

As such, the prevalence of PSC developing after IBD is forecasted to increase to 11.7 by 2027 (95% PI: 10.8, 12.7), rising to 13.3 per 100,000 population when accounting for those who develop IBD after a PSC diagnosis (Table 2 and Fig. 4). The predominant sub-groups living with PSC-IBD in 2027 are estimated to be patients of male sex, and individuals aged 30–44 y with UC; however, the forecasted rate of growth is expected to be similar between sexes, and among those with PSC-CD and PSC-UC (Table 2, Fig. 4, Fig. 5).

**Fig. 6:**
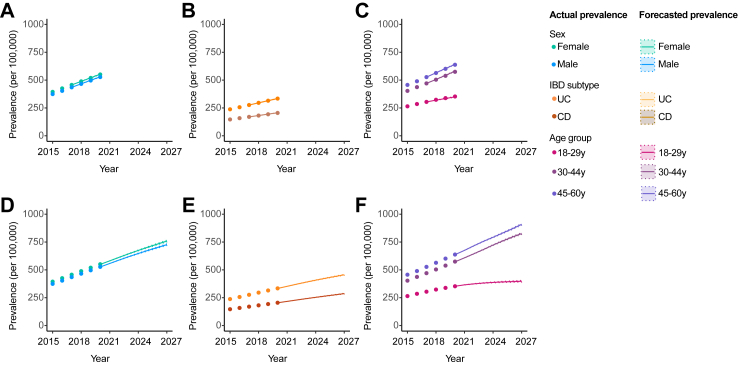
**Actual and forecasted prevalence for IBD by subgroup. (A–C)** The prevalence of IBD alone is shown until the 2020. Years 2017–2020 were used as a calibration set, wherein the actual prevalence (circles) is compared to the estimated prevalence (solid lines) with 95% prediction intervals (dashed lines) forecast from the years prior, stratified by sex **(A)**, IBD subtype **(B)**, and age group **(C)**. In **(D–F)** The future prevalence of PSC-IBD, forecast to 2027 is also shown, stratified by sex **(D)**, IBD subtype **(E)**, and age group **(F)**. The future prevalence of IBD alone, forecast to 2027 is shown using the “adjusted” model as described previously.

In contrast, the estimated prevalence of IBD alone is expected to reach 742.5 per 100,000 population (95% PI: 736.4, 748.0), with an AAPC of 4.7 (95% PI: 4.6–4.8) (Fig. 4). Similar rates of growth are predicted for men and women, and according to IBD subtype (Table 2), and with a plateauing of IBD prevalence among 18–29-year-olds (Fig. 6).

## Discussion

To help overcome the barriers involved in testing (and validating) new therapies for rare disease, it is essential to obtain robust descriptors of disease epidemiology, how this is likely to change, and in particular, capture the principal demographic. However, for the rare liver disease PSC, there is a distinct lack of nationwide population-based data.^18^ Indeed, the lion’s share of insight into temporal trends stem from regional estimates, the local catchment area of specialist centres, and without censoring prevalent cases at the time of liver transplantation. This can lead to selection bias, false estimations of prevalence, and inaccurate sample sizes on which to make downstream inferences. Collaborations like the Global Burden of Disease initiative have enhanced understanding of IBD epidemiology,^19^ yet robust analyses relating to the subgroup of patients with PSC have not been presented.

In this study of whole-of-England data, we show that the prevalence of PSC-IBD is rising, will continue to increase, and that the rate of growth will exceed that of IBD alone. Additionally, regions of heightened PSC-IBD prevalence do not mirror those of peak IBD prevalence; a pattern that is also seen when comparing the incidence of both diseases.^1^ We also find that whilst PSC is a disease that will continue to predominate among young men, the rate of growth in women will parallel, with the main affected demographic being individuals aged 30–44 years. This contrasts to IBD alone, whereby individuals of older age are predicted to make up the majority of patients, with no major differences in prevalence growth between the sexes.

Regional reports from New Zealand, Norway and the Netherlands have also demonstrated an increasing prevalence of PSC.^20^, ^21^, ^22^ This is alongside historic UK primary care data, sampling at most ∼7% of the total population.^23^ However, ours is the first to quantify the rate of growth in PSC-IBD and forecast how disease prevalence is likely to change over time, including by demographic subgroups. Of note, we show that prevalence is likely to increase at a rate of ∼6.5% per year in England.

The rising prevalence of IBD has been shown across Europe, North and Latin America, Oceania; and in newly industrialised countries occupying South and Far East Asia.^9^^,^^19^^,^^24^^,^^25^ As the incidence of IBD in developed nations is steady, the rise in prevalence has likely arisen as incidence outpaces death, with new medical therapies prolonging survival free of clinical events. Indeed, England appears to be in a state of compounding prevalence, wherein IBD incidence has seemingly plateaued,^1^ Indeed, a plateauing in the incidence of adult-onset IBD has been reported across the western world, and is thought to reflect a transition in exposomal exposures.^9^^,^^26^ For instance, stabilisation in antibiotic use across middle–high income countries^27^—a recognised risk factor for the development of IBD,^28^ lower rates of smoking, changes to hygiene measures, and a reduction in the rates of enteric infections.^29^ These findings contrast with the incidence of IBD in children, which is seemingly increasing and may relate to the greater impact of genetic predisposition and potentially increased consumption of refined and ultra-processed foods from a young age.^26^^,^^29^^,^^30^ Importantly, our forecasted rates of growth across England exceed contemporary IBD estimates from North America, but mirror those seen in UK’s devolved nations.^25^ Collectively, this suggests that the prevalence of IBD is growing at a rate of >4% per year across the UK.

Whilst a large proportion of prevalent IBD patients are above the age of 65,^31^ our goal was to compare prevalence against an age-matched group of PSC-IBD patients in whom the incidence of PSC-related clinical events is greatest (i.e., those aged 18–60 years). In so doing, we find that the doubling time of IBD prevalence is estimated to be ∼12 years in this age group, much shorter than reported in studies that encompass older patients.^32^ Reasons for such differences are not known, but it is proposed that environmentally derived, gut microbiotal alterations are a key factor driving the increased incidence in IBD seen early life. Diets containing high levels of fats and refined sugars, improvements to hygiene and sanitation, and greater exposure to antibiotics have been suggested as potential trigger mechanisms.^33^^,^^34^

By contrast to IBD, the absence of licensed medical therapy for PSC means that the rising prevalence is likely a consequence of accelerated incidence.^1^ The fact that PSC-IBD prevalence is increasing at a rate that exceeds IBD alone also supports the presence of additional aetiological drivers,^35^^,^^36^ which need to be studied, tested and confirmed. Increased awareness of PSC amongst physicians who treat IBD is also important, particularly for sub-clinical presentations of disease, given the heightened risks of malignancy. There is also an argument that IBD-specific treatment goals should be directed towards mucosal healing in PSC. This is because persistent inflammation is a risk factor for colorectal cancer,^37^ and of liver-related outcomes following transplantation.^38^, ^39^, ^40^

Indeed, earlier identification of PSC would merit inclusion into annual colorectal cancer and hepatobiliary surveillance programs,^1^^,^^22^^,^^41^ and early access to clinical trials of potentially disease modifying therapy.^8^ Whilst it was not possible to determine whether the threshold for requesting MRI scans has changed over time, the overall number of scans conducted in 2015 and 2020 was similar across England. Moreover, areas of heightened PSC-IBD prevalence did not seemingly mirror areas with greater access and availability of MRI scanners, nor to regions of greater scan activity. It is nevertheless important for future studies, both in IBD and PSC-IBD specifically, to determine how much of the changes in disease epidemiology we observe are a reflection of increased disease awareness by physicians, and/or wider access to imaging.

The HES registry and linked datasets do not house information on the quality of healthcare and accessibility to specialist services, although the latter is subject to ongoing audit.^42^ However, England is home to a wholly publicly funded healthcare system, free from the point of use to all residents. Whilst there is some inevitable variation in care from centre to centre, dedicated IBD specialists are commonplace.^43^ Equally, accredited hepatology centres, whilst fewer in number, are widely distributed across the country,^44^ and the areas of heightened PSC-IBD prevalence did not specifically cluster around any one of England’s six tertiary liver units.

Our study is not without limitation, given the lack of generalisability to countries outside the Northern hemisphere where PSC is reportedly less common. Nevertheless, colleagues from Ontario, Canada also report a rising prevalence of PSC-IBD, at a rate that exceeds growth in IBD alone (Leung and Hirschfield, personal communication). It is nevertheless critically important that we validate our methodology, study findings and forecasting models using administrative healthcare data from other European nations, through multi-centre collaborative initiatives.^22^^,^^45^^,^^46^ Additionally, we chose to quantify PSC and IBD prevalence exclusively in adults above the age of 18 years, and do not present data relating to children living with the disease. Largely, this is a reflection of how access to healthcare data is managed in the UK, with different rights and access permissions for paediatric age groups compared to adults. There are also limitations in using administrative healthcare records when deriving epidemiological estimates of a rare disease such as PSC. Our approach to case finding is reliant on the correctness of clinical coding. Thus, we sought the most homogeneous PSC patient cohort to test a hypothesis; namely whether the prevalence of IBD with PSC differs to that of IBD alone. In so doing, we tried to maximize legitimacy of diagnosis by excluding those with other concomitant liver disorders and including only those patients who had undergone a relevant investigation (or intervention) from which an IBD and PSC diagnosis can be inferred. However, it is plausible that estimates may still underestimate the true prevalence of PSC in England, as approximately 20% of patients do not develop IBD.^47^ Indeed, a major limitation is the fact that it was not possible to identify patients with PSC without IBD, because the accuracy of coding is not validated in this context. Future epidemiologic studies using this data set may therefore benefit from studying the recently introduced ICD11 code for PSC.

From a methodological perspective, we were also unable to provide adjusted prevalence estimates according to race. This is because ONS England population estimates are no longer provided for individual racial or ethnic subgroups split by age and sex. Moreover, given the size of England as a nation, population-level data is presented on a regional scale. Such analysis is too crude and heterogenous to draw any conclusions on the potential contribution of environmental and exposomal risk factors, for which sub-studies exploring regional clustering in densely populated areas would be more appropriate.^48^ Additionally, we had to restrict PSC prevalence estimates to the population already diagnosed with IBD, or who developed IBD within five years after PSC. As we wanted to calculate prevalence on the 1st Jan 2020 using data from 2019 and previous years, for patients diagnosed with PSC at the end of 2019 it is not possible to ‘look ahead’ and see whether they develop IBD at a date after this timeframe. We therefore acknowledge that there are chances we are not capturing a proportion of PSC patients who develop IBD at a much later date.

Whilst our case finding strategy has been tested and externally validated,^49^ it is plausible that some incident cases may have been missed due to a washout between diagnosis and administrative healthcare coding. Or reciprocally, there is a possibility that some incident diagnoses are actually prevalent, due to a delay in clinical coding being applied >12 months after index presentation. However, as demonstrated in the testing step of our forecasting models, the predicted prevalence closely mirrored that of observed prevalence during 2018–2020, providing some evidence of internal validation.

In summary, our data provides nationwide estimates reflecting the current and future landscape of PSC-IBD, showing that the prevalence is rising in England at a rate faster than that predicted by IBD alone. As our administrative healthcare datasets capture every encounter a patient has with secondary care services (including procedure and medication costs), they may be used to inform future health technology assessments, economic impact and for service evaluation, and allow policy makers to develop strategies for high-quality and cost-effective care in rare liver disease.

## Contributors

HC: data extraction, study design, data and statistical analysis including predictive modelling, writing of first draft of the manuscript including figures. JF, MNQ, RC and THI: reviewing of data extracts and critique of subsequent analysis, reviewing of first and subsequent draft of the manuscript, and provision of critical insight needed for revising the manuscript to finalisation. PJT: securing grant funding, study conceptualisation, study design, writing of the first and subsequent drafts of the manuscript including figure revisions, and subsequent manuscript edits through to submission.

## Data sharing statement

This study pertains to data held by the Hospital Episode Statistics (HES) registry. HES data are available to be analysed under licence directly from NHS-England (link: Users, uses and access to Hospital Episode Statistics - NHS England Digital). Individual patient data cannot be provided or shared directly by the authors; however, requests for aggregate data from the HES registry can be made by applying through the Data Access Request Service (link: DARS), and for the specific study dataset thereafter to the University Hospitals Birmingham (UHB), following appropriate governance processes outlined herein: https://researchdata.uhb.nhs.uk/.

Current study data are analysed using assumed consent subject to National data op-out (link: National Data Opt-Out - NHS England Digital). The technical specification for the HES dataset is maintained by NHS England (link: Hospital Episode Statistics Data Dictionary - NHS England Digital). Office for National Statistics (ONS) data can be downloaded direct from the ONS using the ONS API (link: ONS developer Hub - Introduction). All study algorithms and Supplementary information relating to study protocol and statistical analysis plan are documented in the Supplementary documentation.

The National Imaging Data Collection in England and the Diagnostic Imaging Dataset (DID) are publicly available resources, accessible through the links provided.^12^^,^^13^

## Editor note

The Lancet Group takes a neutral position with respect to territorial claims in published maps and institutional affiliations.

## Declaration of interests

All authors have completed the ICMJE disclosure form and declare as follows: PJT receives institutional salary support from the NIHR Birmingham BRC. This paper presents independent research supported by the Birmingham NIHR BRC based at the University Hospitals Birmingham National Health Service Foundation Trust and the University of Birmingham, UK. The views expressed are those of the author(s) and not necessarily those of the National Health Service, the NIHR, or the Department of Health. This study was supported by an unrestricted grant from Gilead sciences, provided to the host institution. No conflicts of interests are declared for the other co-authors.
