# Supplementary material for: Past, current, and future trends in the prevalence of primary sclerosing cholangitis and inflammatory bowel disease across England (2015–2027): a nationwide, population-based study
Source: Lancet Reg Health Eur. 2024 Jul 10;44:101002. doi: 10.1016/j.lanepe.2024.101002 (PMC11296053; doi:10.1016/j.lanepe.2024.101002)
Supplement: Supplementary File 1, Figs. S1–S9, and Tables S1–S6 [file mmc1.docx]

Determining the current and future prevalence of PSC-IBD in England

# Supplementary Materials

## Supplementary File 1: Supplementary Methods

**Study Population and Overview**

This is a nationwide population-based study conducted throughout England, performed via evaluating patient medical records held by the Hospital Episode Statistics (HES) registry and the Office of National Statistics database. HES data encompasses all National Health Service (NHS) Clinical Commissioning Groups in England, detailing every hospital admission, attendance, and investigative and therapeutic procedure undertaken in the NHS. Notably, NHS free health care constitutes 98%–99% of all hospital activity in England,^1^ being available to every British citizen. Patient identification is reliant on the unique 10-digit NHS number ascribed to all registered users of health services in England. Each HES record contains a broad range of data about a patient attending health care services, including information about diagnoses and operations; demographics; and administrative and geographic data such as the dates and route of admission and discharge, patient residential address, location of treatment, and hospital type. No laboratory parameters or medication data are captured. In addition to the HES registry, the Office of Population Censuses and Surveys Classification of Interventions and Procedures version 4, is used to identify all surgical procedures performed throughout the NHS, and the Office of National Statistics to capture the dates, location, and causes of death of all individuals across the United Kingdom.

Extraction of NHS registry data yields fully anonymized records, with no traceable patient identifiable information provided to the researcher. For prior studies in PSC, we have employed case finding through detailed review of all hospital attendances and admissions, outpatient visits, imaging logs (transabdominal ultrasonography scans, magnetic resonance cholangiopancreatography [MRCP] / magnetic resonance imaging (MRI) liver scans, ultrasound-guided and transjugular liver biopsies, endoscopic retrograde cholangiopancreatography, and endoscopic ultrasonography scans), endoscopy reports, theatre reports (bowel resections, liver resections, and liver transplantations [LT]), cancer records (colorectal, bile duct, hepatocellular, gallbladder, and pancreatic), and deaths across the aforementioned time period.^2^

The level of accuracy and completeness of NHS registry data has been presented elsewhere.^3–7^ Notably, in a previous study looking at admissions related to IBD years,^8^ coding was found to be 94.3% accurate, but fell among individuals >60 years of age (detailed below).^8^ Moreover, our own work looking at the incidence of IBD alone and PSC-IBD specifically^2^ also found that the phenotype and year of diagnosis was correct for >90% of patients with UC, Crohn’s disease (CD), PSC-UC and PSC-CD, who attended our host institution between 2006-2016.

Our study comprised three principal aims: (1) to quantify the past and current prevalence of PSC-IBD and IBD alone between the 1^st^ of January 2015 to the 1^st^ of January 2020, (2) construct a well-fitting forecast model, capable of predicting current disease prevalence (1^st^ of January 2018 to the first of January 2020) using epidemiological data from the years prior, and see how well these predicted rates mirror those actually observed in the same time frame; and (3) extrapolate the latter methodology, to construct forecasting models capable of predicting future disease prevalence (between the 1^st^ of January 2021 to the 1^st^ January 2027).

### Data Extraction and Principal Study Period

The principal study period in which annual disease prevalence was determined was between years 2015 and 2020. However, we interrogated the HES registry from the 1^st^ of April 2001 (the first available date of national inpatient data) and the 1st of April 2006 (the first available date of national outpatient data) up until the 31st of December 2019. This is because patients with IBD were only counted in prevalence estimates if there was evidence they had undergone an investigation or practical procedure at some point, that was capable of making a diagnosis (such as compatible imaging, a colonoscopy, or flexible sigmoidoscopy). Similarly, patients with PSC were only counted in prevalence estimates if they underwent an investigation compatible with making a PSC diagnosis (such as a previous magnetic resonance cholangiogram, endoscopic retrograde cholangiogram, or liver biopsy). Patients with concomitant coding for another chronic liver disease at any point were excluded, as previously described (**Supplementary Table 2**).^2^

As per previous descriptors of PSC using HES data, only patients with PSC and concomitant IBD were studied (**Figure 1**), given that coding for PSC without IBD is less accurate.^2^ In so doing, patients with PSC were only counted in prevalence estimates between 2015 and 2020, if there was evidence of an IBD diagnosis recorded within 10 years before PSC diagnosis. Thereafter, we quantified the number of patients who developed IBD up to five years after PSC diagnosis for each geographic region in England in the year 2015, to provide a corrected estimate of PSC-IBD prevalence nationally. The same correction factor was used to estimate the prevalence of patients who developed IBD within five years after PSC diagnosis for subsequent study years and when applying forecasting models. The five-year prospective time frame was chosen because among patients who develop IBD after PSC, the majority do so within this interval.^2^ Importantly, we quantified the number of PSC patients who developed IBD up to five years after PSC diagnosis in 2015 for each geographic region in England separately and used the mean percentage increase in PSC-IBD cases with vs without IBD after PSC to adjust estimated disease prevalence on any given date. A confidence interval for the mean percentage increase was calculated using the t-distribution.

Additionally, the age range for study was prespecified as >/=18 years and </= 60 years’ old. An upper age band was chosen as clinical coding (with regards IBD and PSC) is less accurate in older age.^8,9^ In a previous study looking at admissions related to IBD, coding was found to be 94.3% accurate; but when split by age only 3% of patients aged between 18 and 60 were inaccurately coded compared with 13% of those aged over 60 years. A similar analysis carried out at a neighbouring hospital trust found a miscoding rate in patients aged 18-60 years of 5.0%, but this increased to 25% in patients aged >60 years. Notably, people of younger presenting age are also those who most often experience a PSC-related clinical event, and thus in greatest need of new PSC therapies.^2,10^

**Study Periods for Forecasting Disease Prevalence**

Next, we set out to create models capable of forecasting disease prevalence, initially for a period where the actual prevalence had already been quantified (current prevalence: 1^st^ January 2018 to 2020), and then annually for future years, where prevalence rates are unknown (future prevalence: 1^st^ January 2021 to 2027) (**Supplementary Figure 1**). The statistical methods for forecasting prevalence were adapted from those developed by Keogh et al., for the rare disease cystic fibrosis.^11^

As such, the methods used for forecasting disease prevalence per year require the following information as input:

(1) The number of prevalent cases at the start of each forecasting period (**Figure 1**);

(2) An estimate of how many incident cases of disease occur in each year prior, and an estimation of how many new incident cases will account for prevalent cases in later years;

(3) The age-specific probability of a patient meeting a clinical endpoint event in a given year (liver transplantation or death); i.e. an event that would remove them being classified as a 'prevalent case of PSC’; and

(4) Projections of how the overall population of England is likely to change over time (obtained Office of National Statistics [ONS]-based population projections for England).

First, several models of forecasting were created based on age, sex and IBD type, using prevalence data from 2015 to 2017, alongside incidence and event free survival rates from years prior (**Supplementary Tables 1 and 3**). The forecasted prevalence from each model was then compared to the actual observed prevalence between 2018 and 2020, which had already been quantified in the step entitled “Determining Current Disease Prevalence.”

The method associated with the best fitting model (in which predicted prevalence most closely mirrored actual observed current prevalence between 2018 and 2020) was then extrapolated upon to create models capable of forecasting future disease prevalence between 2021 and 2027.

All statistical analyses were performed using R (R Foundation for Statistical Computing, Vienna, Austria). Full details on steps (2) – (4) are presented in order below.

## *Modelling and estimating incidence*

An estimate of incidence rates, including how these change over time, is needed to forecast future disease prevalence. To this effect, the date of incident IBD diagnosis was defined as either (a) a patient’s first diagnosis with ulcerative colitis (UC), IBD unclassified (IBD-U) or indeterminate colitis (IC), or Crohn’s disease (CD) to ever be documented; or (b) the date of a first diagnostic investigation associated with making an IBD diagnosis – whichever was first.

As 2012 was the first date in which an ICD10 code for indeterminate colitis was introduced, we chose this year as the starting point when estimating the trends in changes of annual incidence rates over time (the latter being necessary to see how changes in incidence affect changes in disease prevalence), and December 31^st^ 2019 as the end date. The reason earlier dates (pre-2012) were not chosen as a starting point for estimating changes in incidence was because we did not want introduction of a new IBD code to affect incidence trend estimates when fitting downstream models to forecast future disease prevalence.

Individuals were counted as incident cases of IBD at the moment they first received a compatible disease code **(Supplementary Table 2)** or underwent the first diagnostic test compatible with making an IBD diagnosis, whichever was coded first. Individuals were counted as an incident case of PSC the moment they received a compatible disease code (**Supplementary Table 2)** or underwent the first diagnostic test compatible with making a PSC diagnosis, whichever was first. Individuals were only counted as prevalent cases after a minimum of 12 months following their first compatible diagnostic investigation, or a minimum of 12 months after they received a diagnosis code in HES (whichever was first) and provided they had not experienced a clinical event that would remove them from being counted as being prevalent.

Notably, patients with an existing (i.e., prevalent) diagnosis of UC or CD in the 10 years prior to 2012, were excluded for estimations of incidence trend data. Additionally, patients undergoing excision of all or part of the bowel in the 10 years prior to incident IBD diagnosis were also excluded. IBD sub-type was defined as that specified on the date of their incident diagnosis. For both PSC-IBD and IBD incident cohorts, patients aged under 10 years or over 60 years at diagnosis, or with unknown sex were excluded (note: information on disease cases in over 11-year-olds in 2020 was required in order to forecast prevalence in over 18-year-olds in 2027). Cases were also excluded from incidence calculations if a sclerosing cholangitis diagnosis was attributed in the 10 years prior to 2012 (i.e. historical prevalent cases). Akin to our methodology for identifying prevalent cases, individuals were counted as incident PSC-IBD cases if they had a concomitant IBD diagnosis recorded in the 10 years prior to PSC diagnosis, or in the five years after; they did not receive a diagnosis of another hepatobiliary disease as previously defined^2^ (PSC with concomitant autoimmune hepatitis was permitted); and had not undergone a liver transplant in the 10 years prior to first PSC diagnosis.

Importantly, estimating trends in future incidence was needed only to develop forecasting models for future prevalence. Thus, to determine the most accurate estimates for incidence (which would subsequently feed into prevalence forecast estimates), three Poisson models were fitted to the counts of incident cases per year for both PSC-IBD and IBD alone:

1. A ‘simple’ model with age category and log(year) as predictors.
2. A ‘conservative’ model with age category as the sole predictor, fitted to only the most recent three years of data, making the assumption that incidence rates are stable over time.
3. An ‘adjusted’ model, which incorporated IBD subtype, age category, sex and log(year-2000). Many different models of this type were fitted, reflecting the possible options for interactions between variables.

The model with the lowest Akaike Information Criterion (AIC) was then chosen.

For all models, the log of the England population size was included as an offset. Mid-year population estimates by year of age and sex were obtained from the Office of National Statistics (ONS).^12^ via the R package monstR.^13^ These models were separately fitted to data on the trends in incidence from December 31^st^ 2012 to December 31^st^ 2016 (used for forecasting current disease prevalence between January 1^st^ 2018 and January 1^st^ 2020, and thereafter comparing with the actual observed prevalence in this timeframe) and then to trends in incidence from December 31^st^ 2012 to 2019 (used for forecasting future disease prevalence between January 1^st^ 2021 and 2027). *Prior to model fitting, age at diagnosis was converted to a categorical variable, with groupings chosen after looking at plots of incidence rate by year of age, to determine age intervals across which the incidence rate varied little. For PSC-IBD these were 10-19y, 20-29y, 30-39y, 40-49y, and 50-60y, while for IBD these were 10-14y, 15-21y, 22-39y, 40-54y, 55-60y.

### *Estimating survival probabilities*

Next, for the PSC-IBD prevalent cohort, we estimated the probability of transplant-free survival to age ‘a’+1 for a patient who has already survived to age ‘a’. We did similarly for the IBD prevalent cohort, but with absolute survival. These probabilities were assumed to be constant through time. In both cases the probabilities were estimated with the use of a flexible parametric survival model.^14,15^ For the purposes of fitting the survival model, the prevalent cohort data was considered left-truncated at 31^st^ Dec 2010 for patients who were diagnosed prior to that date, and right-censored on 31^st^ Dec 2019 for patients who are not recorded to have died (or, in the case of PSC-IBD, received a liver transplant) before that date. Age as a continuous variable was used as the time scale for the survival models and so dates were converted to the inferred age of the patient on that date. As a sensitivity analysis, two models were chosen for proceeding with the prevalence forecasting calculations: a proportional hazards model with 2 internal knots and no additional predictors, and a non-proportional hazards model with 2 internal knots and IBD subtype as a predictor.

### Flexible parametric survival model

The flexible parametric survival models used in this paper model the log cumulative hazard using a restricted cubic spline with two internal knots. The formula for the basic model (with no dependence on additional covariates) is:

$\log\left( -\log\Pr\left( T>a \right) \right)= \gamma_{0}+ \gamma_{1}\log a+\gamma_{2}\nu_{1}(\log a)+ \gamma_{3}\nu_{2}(\log a)$;

where $\Pr\left( T>a \right)$ is the probability of survival beyond age $a$ and $\nu_{j}(x)$ is the $j$ th basis function.^15^ Additionally, we used a model with IBD type (UC vs CD) as a covariate, for which the formula is:

$\log\left( -\log\Pr\left( T>a | I \right) \right)= \gamma_{0}+\beta_{0}I+ \gamma_{1}(I)\log a +\gamma_{2}\nu_{1}(\log a)+ \gamma_{3}\nu_{2}(\log a)$

where $I$ = 1 for UC and $I$ = 0 for CD and $\gamma_{1}\left( I \right)= \gamma_{1}+ \beta_{1}I$. **Supplementary Table 3** shows the parameter estimates ${(\gamma}_{0}, \ldots, \gamma_{3}, \beta_{0}, \beta_{1})$ and 95% confidence intervals when the above models were fitted to PSC-IBD transplant-free survival data and IBD survival data. Knot positions were chosen using the default method in flexsurv (a statistical package in R developed for modelling of survival data),^15^ from quantiles of the log uncensored event times. For the PSC-IBD data these were 2.60, 4.06, 4.30, 4.59 while for IBD they were 1.50, 4.27, 4.40, 4.64.

## *Generating iterative prevalence forecasts*

Forecasts are obtained using similar methods to those by Keogh *et al*.^11^ We initially describe the methods for forecasting using the simple or conservative incidence models and basic survival model. The application of the same methods to the adjusted incidence model and IBD-type specific survival model is a straightforward extension of this. The forecasting routine is described for PSC-IBD but is identical for IBD.

- Let $\varphi_{a, y}$ be the predicted incidence rate per 100,000 members of the population of age $a$ in year $y$. These values are obtained from the fitted Poisson models described in the methods of the main text. Note that these values are constant within age categories (for a particular year).
- Let $\theta_{a}$ be the probability of survival to age $a+1$ conditional on survival to age $a$. These conditional survival probabilities were obtained from the flexible parametric survival models described in the main text.
- Now let $n_{a, y}$ be the number of individuals with PSC-IBD of age $a$ on the 1^st^ January in year $y$. We wish to calculate $n_{a, y+1}$.
- Define the following further quantities:
  - $N_{a, y}$ is the projected England population of age $a$ at the start of year $y$ (taken from the ONS population projections for that year).
  - $m_{a, y}$ is the number of PSC-IBD patients of age $a$ at the start of year $y$ who survive (transplant-free) to the start of year $y+1$. This is a binominal random variable: $m_{a, y} \sim\text{Bin}\left( n_{a, y},\theta_{a} \right)$
  - $q_{a, y}$ is the number of incident cases of PSC-IBD in individuals of age $a$ at the start of year $y$ who go on to survive (transplant-free) to the start of year $y+1$. This is a binominal random variable: $q_{a, y} \sim\text{Bin}\left( N_{a, y},\varphi_{a, y}\theta_{a} \right)$
  - Then, $n_{a, y+1}=$ $m_{a-1, y}+ q_{a-1, y}$.
- Hence given $\varphi_{a, y}, \theta_{a}, N_{a, y}$ for $a\in11, \ldots, 60$ and $y\in2020, \ldots., 2026$ along with $n_{a, 2020}$ for $a\in11, \ldots, 60$, we may calculate the expected value of $n_{a, y}$ for $a\in18, \ldots, 60$ and $y\in2021, \ldots., 2027$ using the expected values of the above binomial random variables.

For the sex and IBD-type specific forecasts, the number of PSC-IBD patients in each subgroup was forecast separately. For example, to calculate the number of men with PSC-UC of age $a$ at the start of year $y+1$, $n_{a, y+1,\text{M,UC}}$, we used $\varphi_{a, y,\text{M, UC}}$ from the adjusted incidence model, $\theta_{a,\text{UC}}$ from the survival model with IBD type, and population projections for males in England ($N_{a, y, M}$). Then: $m_{a, y,\text{M,UC}} \sim\text{Bin}\left( n_{a, y,\text{M,UC}},\theta_{a,\text{UC}} \right), q_{a, y,\text{M,UC}} \sim\text{Bin}(N_{a, y,\text{M}},\varphi_{a, y,\text{M,UC}}\theta_{a\text{,UC}} )$

And

$$n_{a, y+1,\text{M,UC}}=m_{a-1, y,\text{M,UC}}+ q_{a-1, y,\text{M,UC}}$$

## *Projections of England population size*

The ONS 2018-based principal population projections for England split by year of age were downloaded^16^ and used to generate estimates of future population size. These projections are commonly used to identify the future demand for public services in the UK and are based on recently available population size estimates and assumptions about future fertility, mortality and migration.^17^

## Generating 95% Prediction Intervals

95% prediction intervals were constructed by generating 500 random draws of incidence rates and survival probabilities and using each set of incidence rates and survival probabilities to obtain 50 draws of the numbers of incident cases and surviving prevalent cases from binomial distributions. This resulted in a set of 25,000 predictions of prevalence, the 2.5^th^ and 97.5^th^ percentiles of which define an interval within which the actual prevalence is expected to lie with probability 0.95.

### *Obtaining draws for prediction interval calculation*

For incidence rates, 500 draws of $\varphi_{a, y}$ are generated for each age category and year by using a multivariate normal distribution (with mean given by the parameter estimates and estimated variance covariance matrix calculated from the fitted Poisson model) to get draws of parameters for the model, and then calculating an incidence rate for each draw of the parameters and set of covariate values.

For conditional survival probabilities, 500 draws of $\theta_{a}$ are generated similarly by obtaining draws of the parameters in the survival models from a multivariate normal distribution and then calculating the survival function based on each set of parameters.

$N_{a, y}$ is assumed to be known.

For each draw of $\varphi_{a, y}$ and $\theta_{a}$, we obtain 50 draws of $m_{a, y}$ and $q_{a, y}$ by sampling from their respective binomial distributions. This results in 25,000 values of $n_{a+1, y+1}$. In the next iteration one draw is obtained for each value of $n_{a+1, y+1}$ (so that the number of draws in the subsequent years is still 25,000).

## Calculation of average annual percent change

Average annual percent change (AAPC) across multiple years was calculated as the mean of the annual percent changes for each pair of consecutive years. For example, for the period 2020- 2027 there are 7 pairs of consecutive years and so:

$$\text{AAPC}= \frac{\sum_{y=2021}^{2027} \frac{p_{y}}{p_{y-1}}-1}{7}\times100$$

where $p_{y}$ is the prevalence in year $y$.

To calculate prediction intervals for the AAPC, this calculation was done for each of the 25,000 predictions of prevalence and the relevant percentiles of the resulting distribution of AAPC values were taken.

| **Supplementary Table 1: Timelines used for data extraction and analysis** | | | | |
| --- | --- | --- | --- | --- |
| **Study period and related activity** | **Start date** | **End date** | **Comments** |  |
| **Principal study period** | ***01^st^ Jan. 2015*** | ***01^st^ Jan. 2020*** | ***Time period in which past and current prevalence was quantified*** |  |
| (i) In-patient data extraction | 01^st^ Apr. 2001* | 31^st^ Dec. 2019 | Time period in which data was extracted from in-patient records, interrogated to ensure all counted individuals fulfil criteria for diagnosis, and used to differentiate incident vs. prevalent cases. |  |
| (ii) Out-patient data extraction | 01^st^ Apr. 2006** | 31^st^ Dec. 2019 | Time period in which data was extracted from out-patient records, interrogated to ensure all counted individuals fulfil criteria for diagnosis, and used to differentiate incident vs. prevalent cases. |  |
| **Developing and testing different forecast models to predict current prevalence** | ***01^st^ Jan. 2017*** | ***01^st^ Jan. 2020*** | ***Time period in which actual observed current prevalence compared to that forecast from a prediction model*** |  |
| (iii) Determining past trends in event-free survival to inform design and selection of the optimal prevalence forecast model (current prevalence) | 01^st^ Apr. 2001 | 31^st^ Dec. 2016 | Event-free, age-stratified survival rates determined for all cases of PSC-IBD and IBD alone, to help inform design of current prevalence forecast models.  For the purposes of fitting the survival model, the prevalent cohort data was considered left-truncated at 31st Dec 2010 for patients who were diagnosed prior to that date, and right-censored on 31st Dec 2016 for patients who are not recorded to have died (or, in the case of PSC-IBD, received a liver transplant) before that date.*** |  |
| (iv) Determining past trends in disease incidence to inform design and selection of the optimal prevalence forecast model (current prevalence) | 31^st^ Dec. 2012 **** | 31^st^ Dec. 2016 | Trends in disease incidence modelled from 2012 onward, to help inform design of prevalence forecasting models. |  |
| (v) Estimating trends in later incidence and event-free survival rates (using the data from [iii] and [iv]) to inform design and selection of the optimal prevalence forecast model (current prevalence) | 31^st^ Dec. 2017 | 31^st^ Dec. 2019 | Trends in incidence rates and event-free survival estimated in this time period, to refine the development and selection of the optimum prevalence forecast model. |  |
| **Principal forecasting period** | ***1^st^ Jan 2021*** | ***1^st^. Jan. 2027*** | ***Time period in which future prevalence was forecasted*** |  |
| (vi) Determining past trends in event-free survival to inform design of prevalence forecast model (future prevalence) | 01^st^ Apr. 2001 | 31^st^ Dec. 2019 | Event-free, age-stratified survival determined for all cases of PSC-IBD and IBD alone within this time period, to help inform design of future prevalence forecast models.  For the purposes of fitting the survival model, the prevalent cohort data was considered left-truncated at 31st Dec 2010 for patients who were diagnosed prior to that date, and right-censored on 31st Dec 2019 for patients who are not recorded to have died (or, in the case of PSC-IBD, received a liver transplant) before that date.*** |  |
| (vii) Determining past trends in disease incidence needed to inform design of prevalence forecast models (future prevalence) | 31^st^ Dec. 2012 **** | 31^st^ Dec. 2019 | Trends in disease incidence modelled from 2012 onward, to help inform design of future prevalence forecast models. |  |
| (viii) Estimating trends in later incidence and event-free survival rates (using data from [vi] and [vii] above) to inform design of prevalence forecast model (future prevalence) | 31^st^ Dec. 2020 | 31^st^ Dec. 2026 | Trends in future incidence rates and event-free survival estimated, to inform help inform design of future prevalence forecast models. |  |
| * Indicates the first available date of nationwide records for in-patients.  ** Indicates the first available date of nationwide records for out-patients.  *** Left truncation at 2010 was performed, to allow all PSC patients to have historical years’ data for review, in order to ensure they meet eligibility criteria.  **** The year 2012 was chosen as a starting point for capturing and modelling trends in disease incidence, as this was the year in which a new ICD10 code for IBD unclassified (indeterminate colitis) was introduced. | | | | |

| **Supplementary Table 2: Inclusion and exclusion codes to facilitate case finding and extraction^1^ *** | | | |
| --- | --- | --- | --- |
| **Disease** | **Inclusion codes** | **Exclusion codes** | **Notes on criteria** |
| Ulcerative colitis ** | K51 | K50, K52.3  B16-17, K70-77 (but not K75.4) | Patients with Crohn’s disease or IBD-unclassified diagnosis (concomitant or sequentially) move to the IBD-unclassified category |
| Crohn’s disease ** | K50 | K51, K52.3  B16-17, K70-77 (but not K75.4) | Patients with ulcerative colitis disease or IBD-unclassified diagnosis (concomitant or sequentially) move to the IBD-unclassified category |
| IBD-unclassified ** | K52.3; or concomitant / sequential K50 and K1 | B16-17, K70-77 (but not K75.4) | Also includes any patient with multiple types of IBD diagnoses (concomitantly and/or sequentially-K51 and/or K50) |
| Primary sclerosing cholangitis (PSC) ** | K83.0 | B16-17, K70-77 (but not K75.4) |  |
| Case finding strategy extrapolated from that presented by Trivedi et al.^1^  * ICD10 dictionary can be located through the hyperlink: [https://icd.who.int/browse10/2016/en#](https://icd.who.int/browse10/2016/en) .  ** All included patients with IBD were required to have undergone imaging and / or endoscopic investigations compatible with making a diagnosis of IBD.  *** Only PSC patients with IBD were included. All PSC-IBD patients were required to have undergone imaging and / or endoscopic and/or histological investigations compatible with making a PSC diagnosis. | | | |

| **Supplementary Table 3: Parameter estimates for flexible parametric survival models** | | | | | | | | |
| --- | --- | --- | --- | --- | --- | --- | --- | --- |
|  | **Basic model, PSC-IBD** | | **IBD type model, PSC-IBD** | | **Basic model, IBD** | | **IBD type model, IBD** | |
| **Parameter** | **Estimate** | **95% CI** | **Estimate** | **95% CI** | **Estimate** | **95% CI** | **Estimate** | **95% CI** |
| $\boldsymbol{\gamma}_{\boldsymbol{0}}$ | -7.9 | (-11.5, -4.3) | -8.4 | (-12.3, -4.6) | -7.7 | (-8.4, -7.1) | -7.0 | (-7.4, -6.5) |
| $\boldsymbol{\gamma}_{\boldsymbol{1}}$ | 2.0 | (1.0, 3.1) | 2.2 | (1.1, 3.3) | 0.2 | (0.0, 0.4) | 0.1 | (-0.1, 0.2) |
| $\boldsymbol{\gamma}_{\boldsymbol{2}}$ | 9.3 | (7.1, 11.5) | 9.1 | (7.0, 11.3) | 18.7 | (16.5, 20.9) | 17.9 | (15.8, 20.1) |
| $\boldsymbol{\gamma}_{\boldsymbol{3}}$ | -16.5 | (-20.0, -13.2) | -16.3 | (-19.7, -12.9) | -32.4 | (-35.7, -29.1) | -31.1 | (-34.4, -27.8) |
| $\boldsymbol{\beta}_{\boldsymbol{0}}$ |  |  | 0.7 | (-1.1, 2.4) |  |  | -2.2 | (-2.9, -1.5) |
| $\boldsymbol{\beta}_{\boldsymbol{1}}$ |  |  | -0.2 | (-0.6, 0.2) |  |  | 0.4 | (0.3, 0.6) |
| The flexible survival models represent a transformed survival curve. Parameters γ0 to γ3 represent symbols of the regression coefficients and describe the basic shape of transformed survival curves using a natural cubic spline. Parameters *β* _0_ and *β* _1_ describe the age dependent log hazard ratio between the two types of inflammatory bowel disease: Crohn’s disease (CD) and ulcerative colitis (UC). The formula for the basic survival model (with no dependence on additional covariates) is: $\log\left( -\log\Pr\left( T>a \right) \right)= \gamma_{0}+ \gamma_{1}\log a+\gamma_{2}\nu_{1}(\log a)+ \gamma_{3}\nu_{2}(\log a)$; where $\Pr\left( T>a \right)$ is the probability of survival beyond age $a$ and $\nu_{j}(x)$ is the $j$ th basis function. The survival model with IBD type (UC vs CD) as a covariate, for which the formula is: $\log\left( -\log\Pr\left( T>a \vert I \right) \right)= \gamma_{0}+\beta_{0}I+ \gamma_{1}(I)\log a +\gamma_{2}\nu_{1}(\log a)+ \gamma_{3}\nu_{2}(\log a)$; where $I$ = 1 for UC and $I$ = 0 for CD and $\gamma_{1}\left( I \right)= \gamma_{1}+ \beta_{1}I$. | | | | | | | | |

| **Supplementary Table 4: Demographics of prevalent PSC-IBD cases between 2015-2020 (adjusted)*** | | | | | | | |
| --- | --- | --- | --- | --- | --- | --- | --- |
| **Year** | | **2015** | **2016** | **2017** | **2018** | **2019** | **2020** |
| **Group** | |  |  |  |  |  |  |
|  | All | 1550 ** | 1719 | 1902 | 2065 | 2231 | 2393 |
| Sex | Male | 1053 (68%) | 1149 (67%) | 1252 (66%) | 1362 (66%) | 1455 (65%) | 1544 (65%) |
|  | Female | 497 (32%) | 570 (33%) | 650 (34%) | 703 (34%) | 776 (35%) | 849 (35%) |
| IBD type | CD | 330 (21%) | 358 (21%) | 408 (21%) | 452 (22%) | 499 (22%) | 546 (23%) |
|  | UC | 1220 (79%) | 1361 (79%) | 1494 (79%) | 1613 (78%) | 1732 (78%) | 1847 (77%) |
| Age | 18-29y | 431 (28%) | 491 (29%) | 537 (28%) | 571 (28%) | 605 (27%) | 643 (27%) |
|  | 30-44y | 480 (31%) | 555 (32%) | 629 (33%) | 702 (34%) | 777 (35%) | 872 (36%) |
|  | 45-60y | 639 (41%) | 673 (39%) | 736 (39%) | 792 (38%) | 849 (38%) | 878 (37%) |
| Race | White | 1321 (85%) | 1460 (85%) | 1603 (84%) | 1734 (84%) | 1871 (84%) | 1988 (83%) |
|  | South Asian | 99 (6%) | 114 (7%) | 127 (7%) | 140 (7%) | 149 (7%) | 163 (7%) |
|  | Black | 58 (4%) | 63 (4%) | 70 (4%) | 74 (4%) | 78 (3%) | 82 (3%) |
|  | Mixed/Other | 38 (2%) | 40 (2%) | 54 (3%) | 61 (3%) | 65 (3%) | 75 (3%) |
|  | Not declared | 34 (2%) | 42 (2%) | 48 (3%) | 56 (3%) | 68 (3%) | 85 (4%) |
| Characteristics of prevalent cases of PSC-IBD patients are shown for the 1^st^ of January each calendar year.  * Only patients who have had (a) undergone confirmatory investigations to make a diagnosis of PSC and IBD, (b) who have NOT had a concomitant other liver disease diagnosis, undergone a liver transplant, or died; and (c) who have an IBD diagnosis prior to or at the time of PSC diagnosis are included.  ** The number of PSC patients after including those who develop IBD within five years after the start of our principal study period (year 2015) is *n* = 1757. | | | | | | | |

| **Supplementary Table 5: Demographics of prevalent IBD alone cases between 2015-2020 (adjusted)*** | | | | | | | |  |  |
| --- | --- | --- | --- | --- | --- | --- | --- | --- | --- |
| **Year** | | **2015** | **2016** | **2017** | **2018** | **2019** | **2020** | |  |
| **Group** | |  |  |  |  |  |  | |  |
|  | All | 119,452 | 129,605 | 139,833 | 150,029 | 159,779 | 169,547 | |  |
| Sex | Male | 58,067 (49%) | 63,058 (49%) | 68,186 (49%) | 73,211 (49%) | 78,036 (49%) | 83,009 (49%) | |  |
|  | Female | 61,385 (51%) | 66,547 (51%) | 71,647 (51%) | 76,818 (51%) | 81,743 (51%) | 86,538 (51%) | |  |
| IBD type | CD | 45,568 (38%) | 49,445 (38%) | 53,223 (38%) | 57,025 (38%) | 60,713 (38%) | 64,474 (38%) | |  |
|  | UC | 73,884 (62%) | 80,160 (62%) | 86,610 (62%) | 93,004 (62%) | 99,066 (62%) | 105,073 (62%) | |  |
| Age | 18-29y | 22,953 (19%) | 24,779 (19%) | 26,326 (19%) | 27,849 (19%) | 28,904 (18%) | 29,917 (18%) | |  |
|  | 30-44y | 43,675 (37%) | 47,358 (37%) | 50,991 (36%) | 54,869 (37%) | 58,979 (37%) | 63,406 (37%) | |  |
|  | 45-60y | 52,824 (44%) | 57,468 (44%) | 62,516 (45%) | 67,311 (45%) | 71,896 (45%) | 76,224 (45%) | |  |
| Race | White | 100,234 (84%) | 108,030 (83%) | 115,694 (83%) | 123,072 (82%) | 129,870 (81%) | 136,399 (80%) | |  |
|  | South Asian | 8110 (7%) | 8790 (7%) | 9519 (7%) | 10224 (7%) | 10972 (7%) | 11669 (7%) | |  |
|  | Black | 2054 (2%) | 2274 (2%) | 2450 (2%) | 2665 (2%) | 2875 (2%) | 3066 (2%) | |  |
|  | Mixed/Other | 3145 (3%) | 3559 (3%) | 3985 (3%) | 4409 (3%) | 4840 (3%) | 5257 (3%) | |  |
|  | Not declared | 5909 (5%) | 6952 (5%) | 8185 (6%) | 9659 (6%) | 11222 (7%) | 13156 (8%) | |  |
| Characteristics of prevalent cases of IBD without PSC are shown for the 1^st^ of January each calendar year.  * Only patients who have had (a) undergone confirmatory investigations to make a diagnosis of IBD, (b) who have NOT had a concomitant other liver disease diagnosis, undergone a liver transplant, or died; and (c) who were never diagnosed with PSC. | | | | | | | | | |

| **Supplementary Table 6: Relative risks of PSC among patients living with IBD over time** | | | |
| --- | --- | --- | --- |
| **Year** | **IBD overall** | **UC** | **Crohn’s disease** |
| **2015** | (Reference) | (Reference) | (Reference) |
| **2016** | 1·02 (95% CI: 0·95, 1·09) | 1·03 (95% CI: 0·95, 1·11) | 0·99 (95% CI: 0·86, 1·16) |
| **2017** | 1·05 (95% CI: 0·98, 1·12) | 1·04 (95% CI: 0·97, 1·13) | 1·06 (95% CI: 0·92, 1·22) |
| **2018** | 1·06 (95% CI: 0·99, 1·13) | 1·04 (95% CI: 0·97, 1·13) | 1·09 (95% CI: 0·94, 1·23) |
| **2019** | 1·08 (95% CI: 1·01, 1·15) | 1·05 (95% CI: 0·98, 1·14) | 1·13 (95% CI: 0·99, 1·30) |
| **2020** | 1·09 (95% CI: 1·02, 1·16) | 1·06 (95% CI: 0·99, 1·14) | 1·17 (95% CI: 1·02, 1·34) |
| 95% CI, 95% confidence interval; IBR, inflammatory bowel disease; UC, ulcerative colitis. | | | |

| **Revised Supplementary Figure 1: Statistical analysis plan** |
| --- |
| **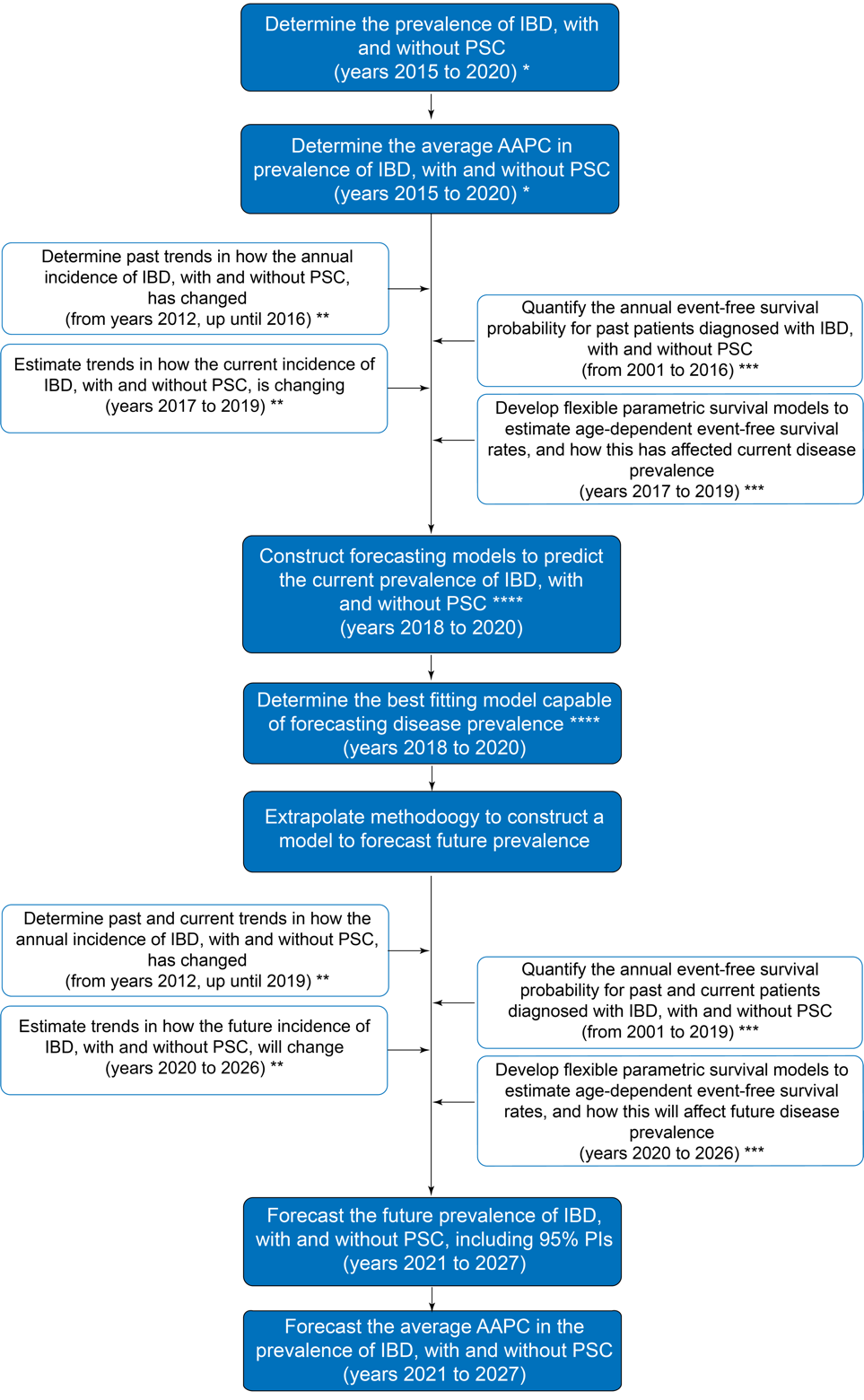** |
| An overview of the statistical analysis plan, designed *a priori*, is shown in the presented infographic.  * Past and current prevalence, including AAPC rates determined as per the infographic presented in **Main Figure 1**.  ** Trends in estimated incidence rates over time were estimated only to inform the design of prevalence forecasting models. December 31^st^ 2012 was chosen as the first year for estimate incidence trend data, due to the introduction of a new code for IBD unclassified / indeterminate colitis.  *** Age-specific event-free survival estimates were determined only to inform the design of prevalence forecasting models. Data from all prevalent patients from April 1^st^ 2001 onward were used; however, for the purposes of fitting the survival model, the prevalent cohort data was considered left-truncated at 31^st^ Dec 2010 for patients who were diagnosed prior to that date, and right-censored on 31^st^ Dec 2019 for patients who are not recorded to have died (or, in the case of PSC-IBD, received a liver transplant) before that date.  **** Three different models of forecasting disease prevalence were constructed, based on age alone; age and sex; and age, sex and IBD-type. Before selecting the optimum method of forecasting future prevalence of disease between January 1^st^ 2021 and 2027, each model was tested in its ability to forecast current disease prevalence between January 1^st^ 2018 and 2020 (i.e., a time-period where the actual disease prevalence had already been quantified), using prevalence trend data from January 1^st^ 2015 to 2017, incidence trend data from December 2012 to 2016, and event-free survival data from April 1^st^ 2010 to December 31^st^ 2016. The method that generated the best fitting prevalence forecast model, in which predicted disease prevalence between 2018 and 2020 most closely mirrored actual disease prevalence between 2018 and 2020, was then selected and applied to create a model for forecasting disease prevalence between 2021 and 2027. |

| **Supplementary Figure 2: Temporal development of IBD among patients with PSC** |
| --- |
| 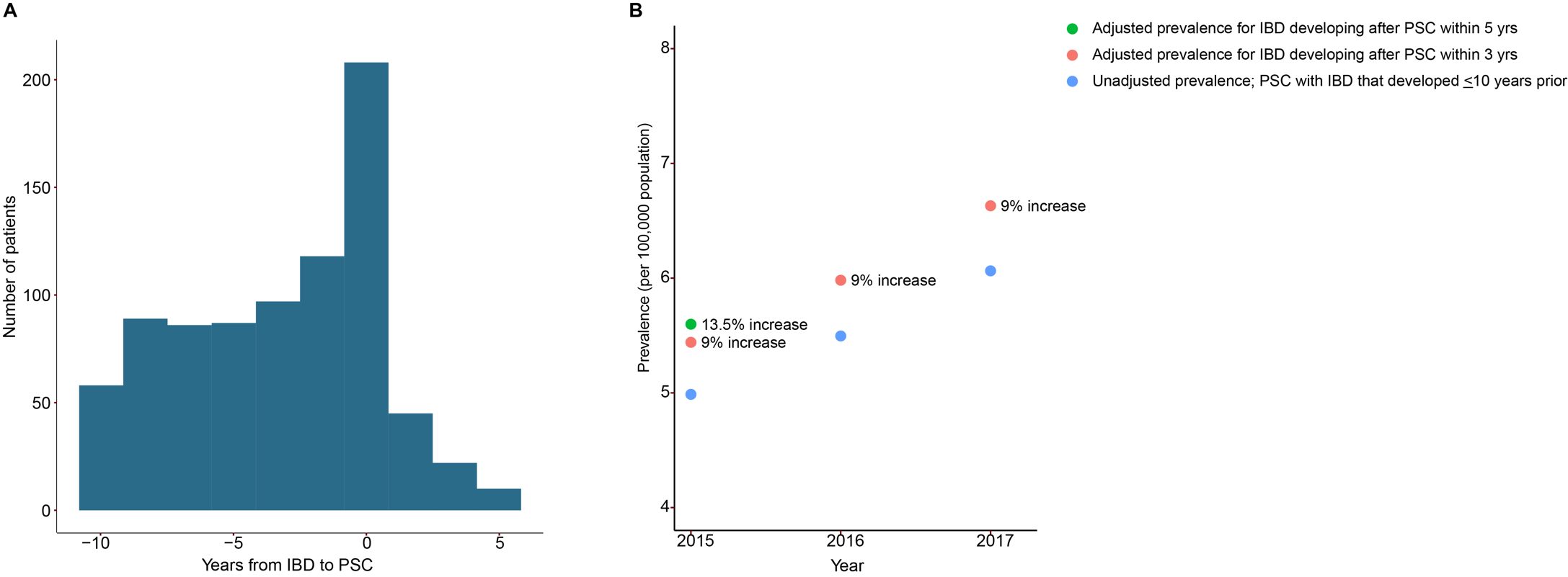 |
| (**A**) The histogram shows a representative sample of patients (n=820) diagnosed with PSC in England prior to the start of prevalence being determined in our study (2011-2014). The majority were diagnosed with IBD up to 10 years before (<0 years on the X axis) or 5 years after (>0 years on the X axis) after the PSC start date. (**B**) The dot plot shows data from years 2015-2017, indicating the prevalence of PSC-IBD between 2015-2017: blue circles indicate the prevalence of patients with an IBD diagnosis up to 10 years prior to PSC, red circles indicate the increased prevalence when including patients diagnosed with IBD up to three years after PSC, and green circles indicate the increased prevalence when including patients diagnosed with IBD up to five years after PSC. |

| **Supplementary Figure 3: MRI scan rates across England over time** |
| --- |
| **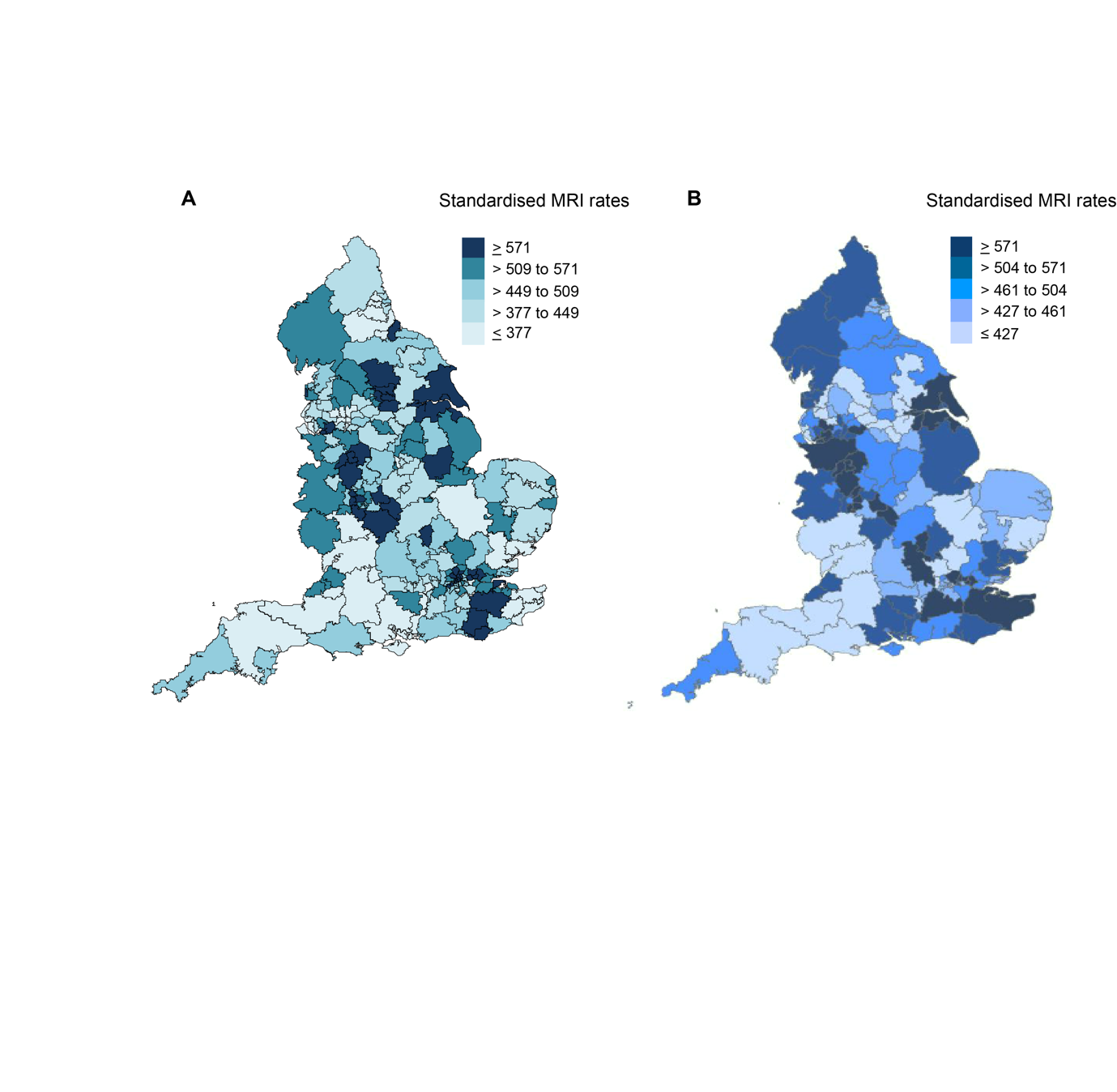** |
| Choropleth maps indicating the number of MRI scans performed across each health board in England, standardised per 100,000 population. Hierarchical colour coding indicates the regions of greatest (dark) to lowest scan activity (light). Data presented for the year 2015 in (A), and 2020 in (B). 3,085,065 and 3,008,970 MRI scans were performed in 2015 and 2020, respectively, across England. The presented infographics contain National Statistics and National Ordnance Survey data, which are subject to Crown copyright and database right. |

| **Supplementary Figure 4: Distribution of MRI scanners across England** |
| --- |
| **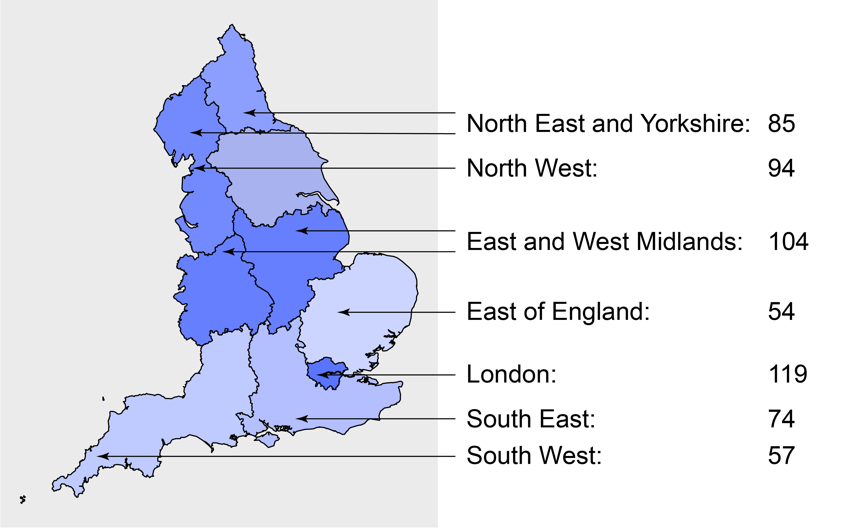** |
| Choropleth map detailing the number of MRI scanners in England by region. Hierarchical colour coding indicates the regions of greatest (dark) to lowest (light) number of MRI scanners; actual numbers provided in parenthesis. Data extracted from the National Imaging Data Collection Asset in England. |

| **Supplementary Figure 5: Incident cases of PSC-IBD per 100,000 population by year, age and IBD subgroup** |
| --- |
| 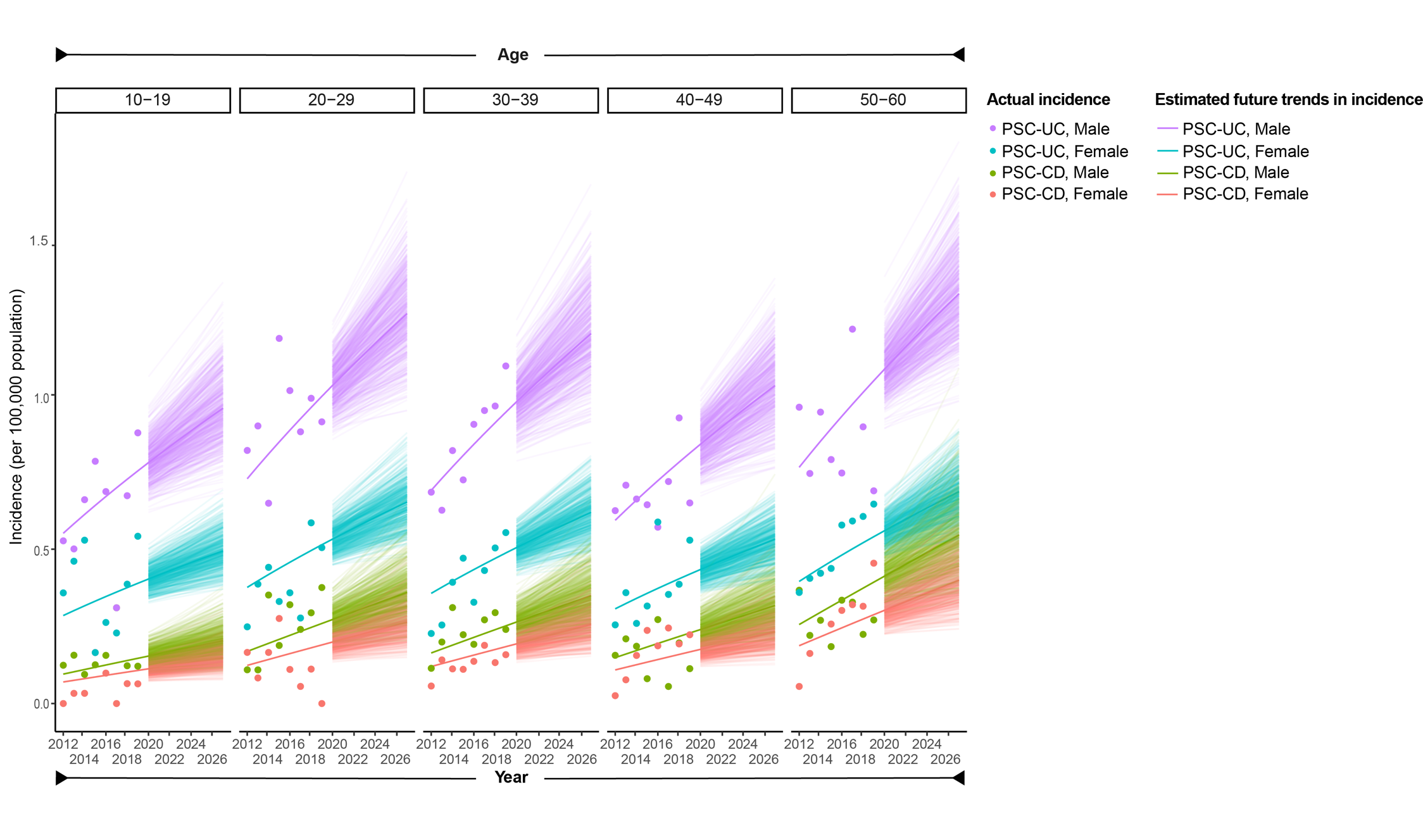 |
| Coloured dots show observed counts of incident cases (between December 31^st^ 2012 and December 31^st^ 2019) and thicker solid lines show the model fit. The model used is the “adjusted model.” There were 2283 incident cases of PSC-IBD between 1st Jan 2012 and 31st Dec 2019. These are split by IBD type and sex and ten-year age brackets. The overall incidence rate for PSC-IBD in 2012 was 0.62 per 100,000 population, rising to 0.91 per 100,000 population in 2019. The faint lines that follow show model predictions generated from 500 separate draws of the model parameters, out to the 31^st^ of December 2026. |

| **Supplementary Figure 6: Transplant-free survival in PSC-IBD according to age** |
| --- |
| 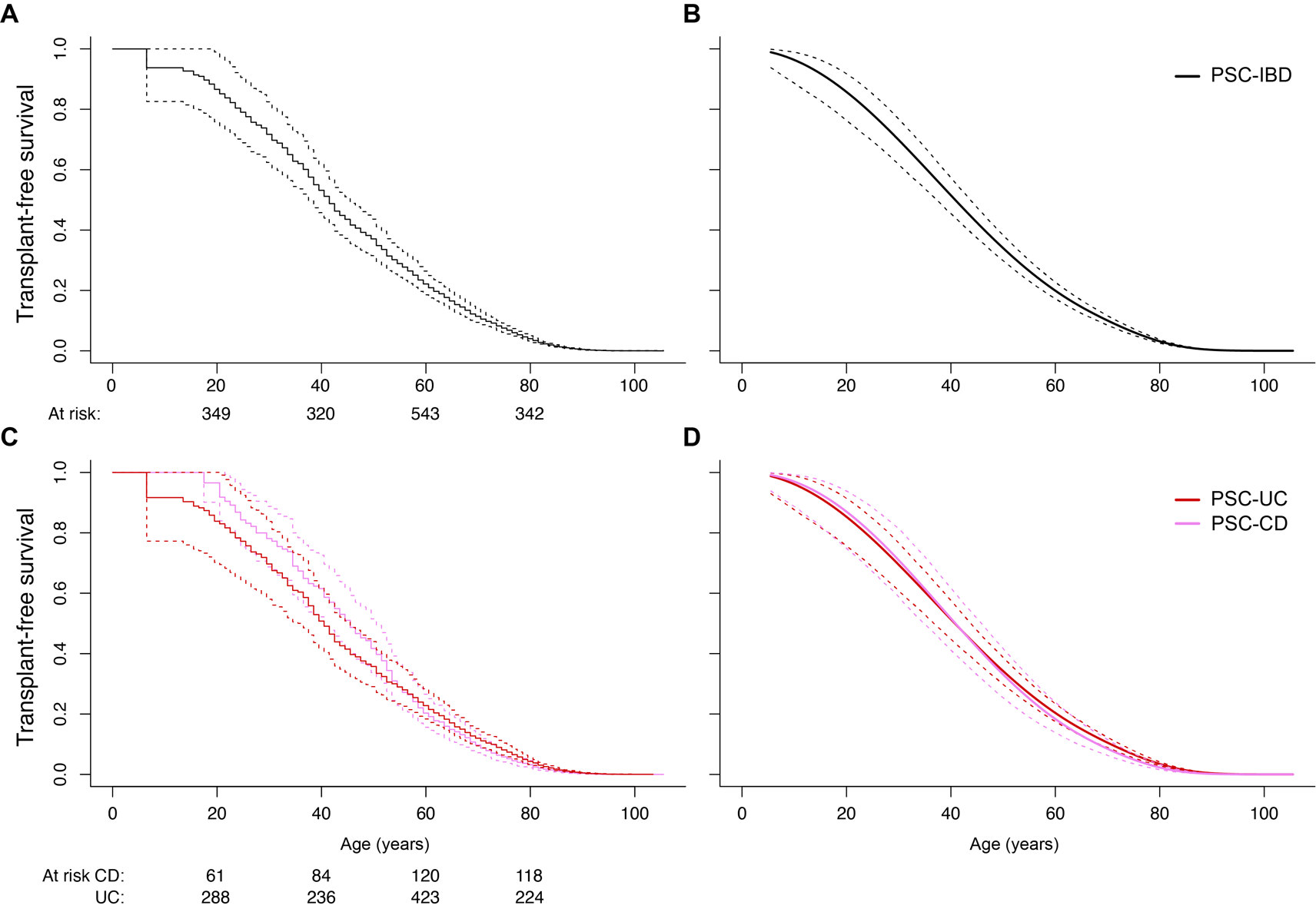 |
| (**A**) Kaplan Meier transplant-free survivorship estimates showing transplant-free survival with increasing age, among patients diagnosed with PSC and IBD, prior to the 31^st^ of December 2016 across England. At risk values indicate numbers of prevalent patients for a single year of age as shown on the X-axis. (**B**) Flexible parametric survival model fit, with proportional hazards and two knots. This model is fitted to the data underlying the prior Kaplan Meier curve, as a model for event-free survival, up to the year 2019. (**C**) Kaplan-Meier transplant-free survivorship estimates among patients diagnosed with PSC and IBD, up to the 31^st^ of December 2016 across England, split by IBD type and according to age. At risk values indicate numbers of prevalent patients for a single year of age as shown on the X-axis. (**D**) Flexible parametric survival model fit, with non-proportional hazards, two knots and IBD type as a predictor, to the data underlying the prior Kaplan Meier curve, as a model for event-free survival, up to the year 2019. Dotted lines indicate 95% confidence intervals. At risk numbers for the Kaplan Meier plots according to age (incident PSC-IBD cases) are given below the x-axis. |

| **Supplementary Figure 7: Incident cases of IBD per 100,000 population by year, age and IBD subgroup** |
| --- |
|  |
| 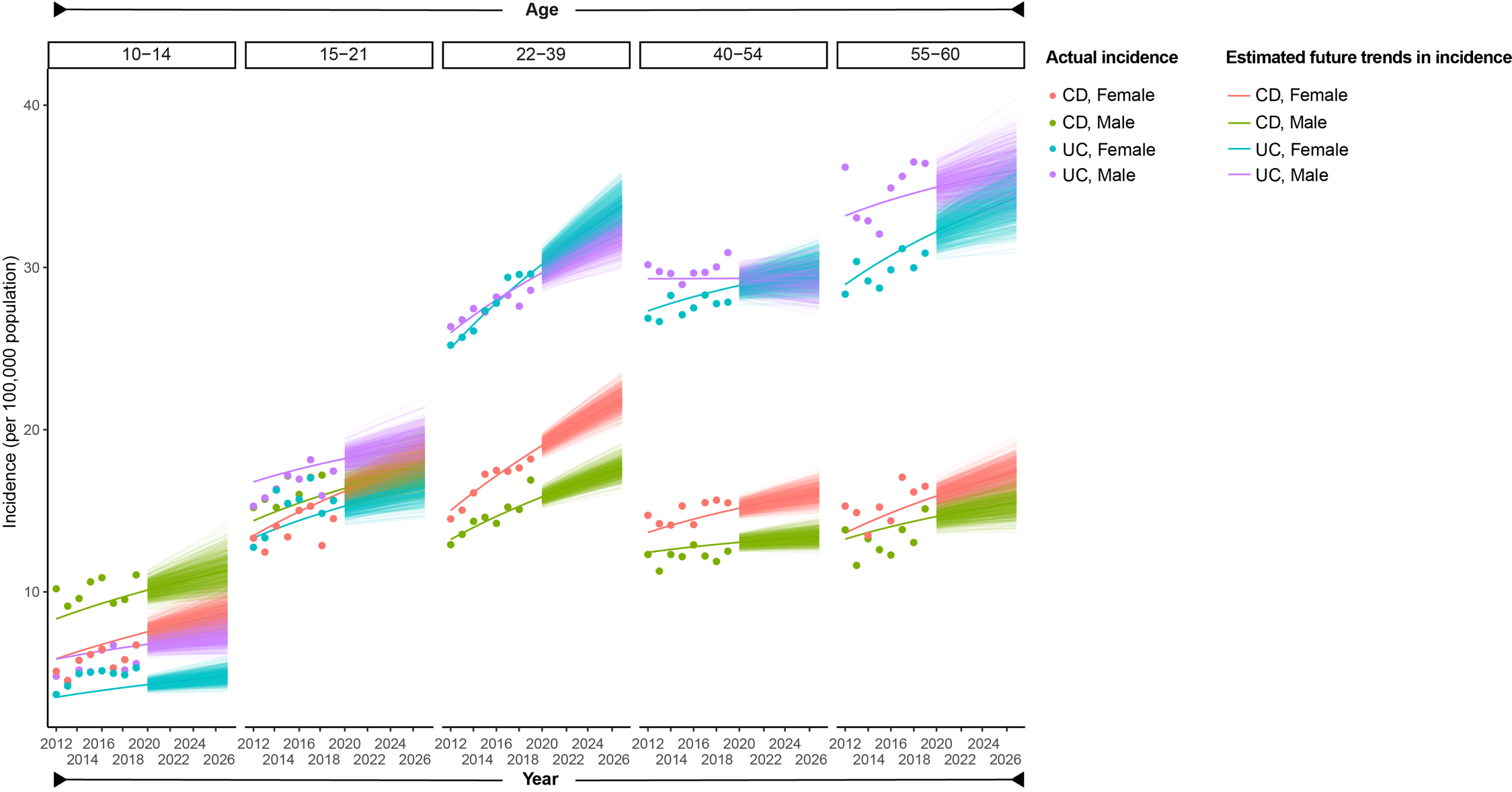  Coloured dots show observed counts of incident cases (between December 31^st^ 2012 and December 31^st^ 2019) and thicker solid lines show the model fit. The model used is the “adjusted model.” There were 112,989 incident cases of IBD captured between 1st Jan 2012 and 31st Dec 2019. These are split by IBD type and sex and ten-year age brackets. The overall incidence rate for IBD (UC & CD) in 2012 was 37.2 per 100,000 population and 41.2 per 100,000 population in 2019. The faint lines that follow show model predictions generated from 500 separate draws of the model parameters, out to the 31^st^ of December 2026. |

| **Supplementary Figure 8: All-cause mortality in IBD according to age** |
| --- |
| 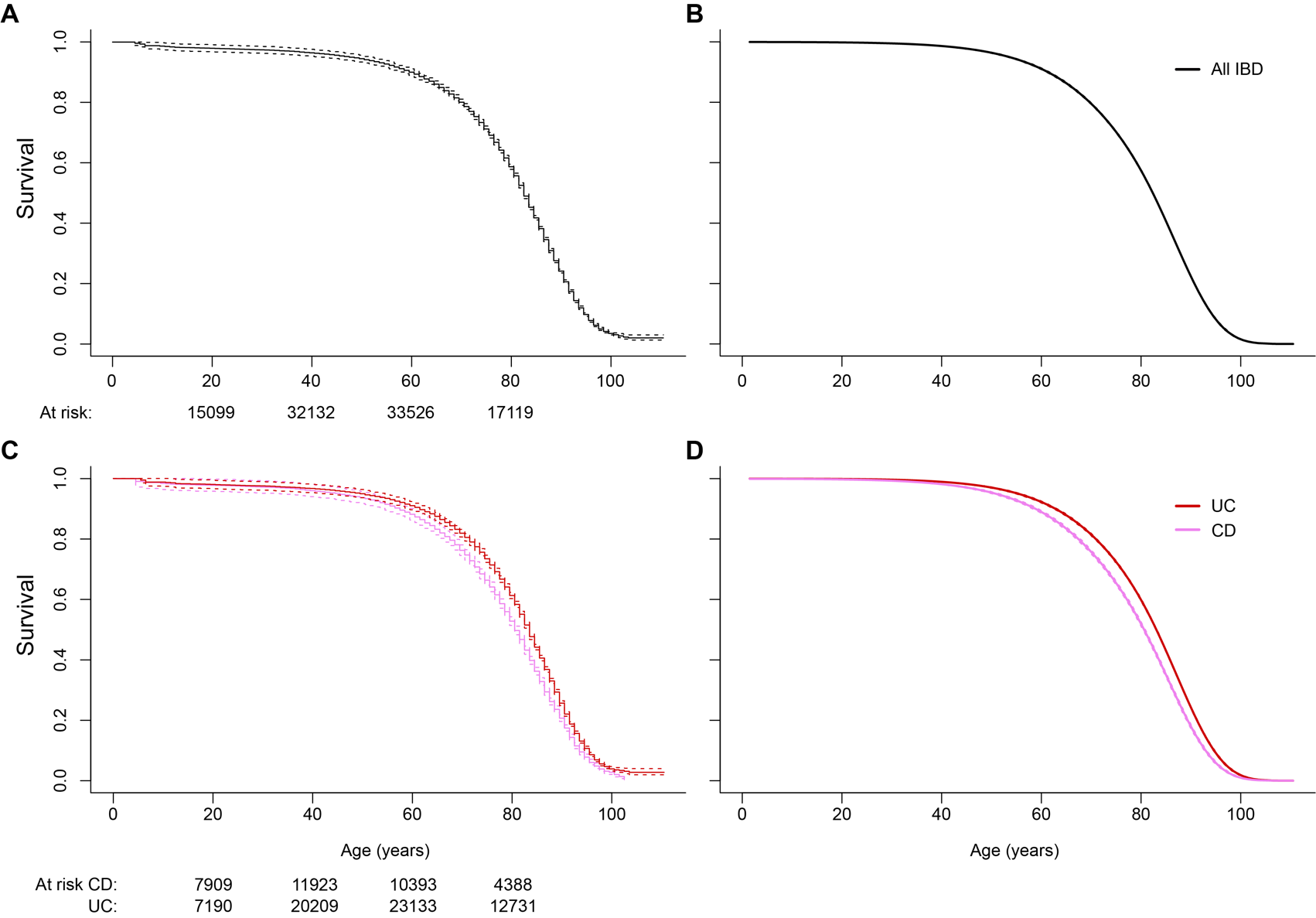  (**A**) Kaplan Meier transplant-free survivorship estimates (all-cause mortality) according to age, among patients diagnosed with IBD alone, identified up to the 31^st^ of December 2016, across England. At risk values indicate numbers of prevalent patients for a single year of age as shown on the X-axis. (**B**) Flexible parametric survival model fit, with proportional hazards and two knots. This model is fitted to the data underlying the prior Kaplan Meier curve, as a model for event-free survival, up to the year 2019. (**C**) Kaplan-Meier survivorship estimates (all-cause mortality) for patients diagnosed with IBD up to the 31^st^ of December 2016 across England, split by IBD type and according to age. At risk values indicate numbers of prevalent patients for a single year of age as shown on the X-axis. (**D**) Flexible parametric survival model fit, with non-proportional hazards, two knots and IBD type as a predictor, fitted to the data underlying the prior Kaplan Meier curve, as a model for event-free survival, up to the year 2019. Dotted lines indicate 95% confidence intervals. At risk numbers for the Kaplan Meier plots according to age (incident IBD cases) are given below the x-axis. |
| **Supplementary Figure 9: Actual and forecasted prevalence for PSC-IBD and IBD alone, based on alternative incidence and mortality models**  **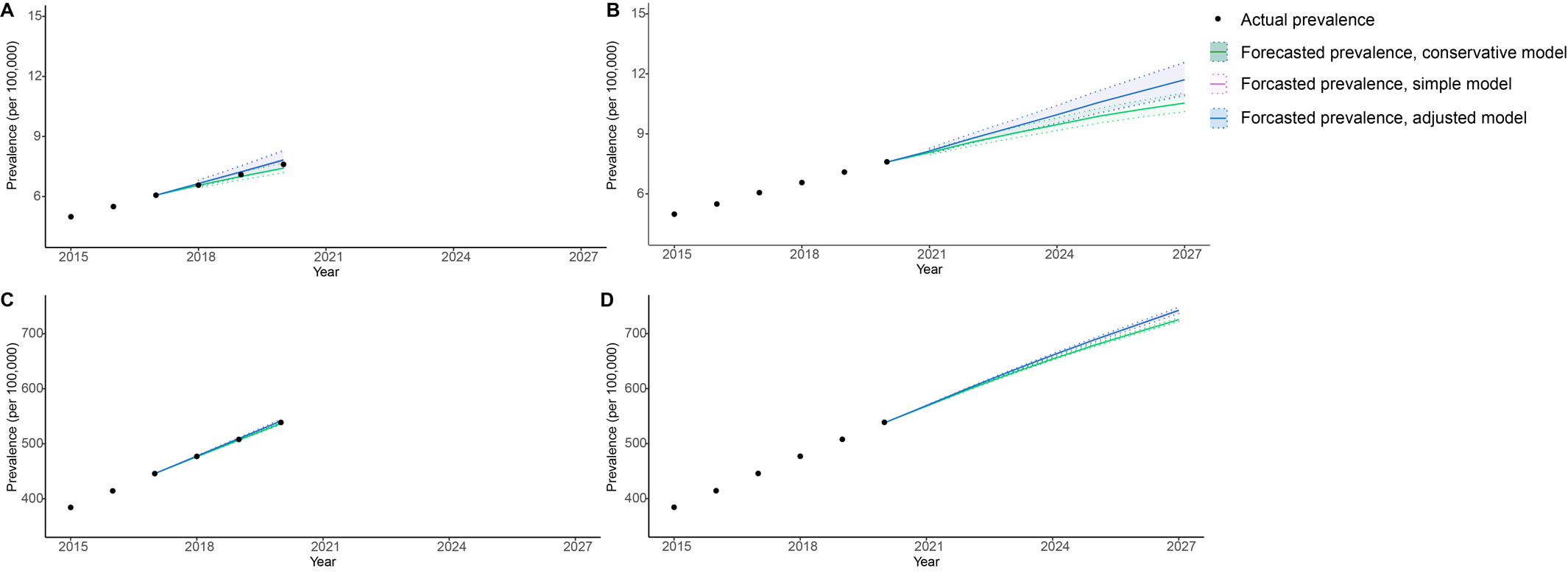** |
| The past, current and future prevalence of PSC-IBD and IBD alone are shown. In panel **(A)** the actual observed prevalence of PSC-IBD is shown until the 2020 (black circles). The actual observed prevalence in years 2018-2020 (current prevalence) were then compared to values predicted from a forecasting model, derived from past prevalence data, different estimated incidence models, and estimated mortality rates: a “simple” model (lilac), with only age category and log(year – 2000) as predictors; a “conservative” model (green), with age category as the sole predictor, fitted to only the most recent three years of data, making the assumption that incidence rates are stable over time; and an “adjusted” model, which incorporated IBD subtype, age category, sex and log(year-2000). Many different models of this type were fitted, reflecting the possible options for interactions between variables. The best fitting model, in which forecast current prevalence most closely mirrored actual observed prevalence between 2018 to 2020, was then determined. In panel **(B**), the three different models are shown in their ability to forecast the future prevalence of PSC-IBD, up until to 2027. **(C)** The current and forecasted prevalence of IBD alone is shown, using each of the three models as described previously. **(D**) The future prevalence of IBD alone, forecast to 2027 with each of the three models is shown. Note: the model with the lowest Akaike Information Criterion (AIC) was then chosen and presented in main body text thereafter; specifically, the “adjusted” model. |

## Supplementary References

1. Herbert, A. Data Resource Profile: Hospital Episode Statistics Admitted Patient Care (HES APC) | International Journal of Epidemiology | Oxford Academic. https://academic.oup.com/ije/article/46/4/1093/3072145.

2. Trivedi, P. J. *et al.* Effects of Primary Sclerosing Cholangitis on Risks of Cancer and Death in People With Inflammatory Bowel Disease, Based on Sex, Race, and Age. *Gastroenterology* **159**, 915–928 (2020).

3. Holt, P. J. E., Poloniecki, J. D. & Thompson, M. M. Multicentre study of the quality of a large administrative data set and implications for comparing death rates. *BJS* **99**, 58–65 (2012).

4. Burns, E. M. *et al.* Systematic review of discharge coding accuracy. *J. Public Health* **34**, 138–148 (2012).

5. Britton, A. *et al.* Validating self-reported strokes in a longitudinal UK cohort study (Whitehall II): Extracting information from hospital medical records versus the Hospital Episode Statistics database. *BMC Med. Res. Methodol.* **12**, 83 (2012).

6. Vallance, A. E. *et al.* Impact of hepatobiliary service centralization on treatment and outcomes in patients with colorectal cancer and liver metastases. *Br. J. Surg.* **104**, 918–925 (2017).

7. Parisi, R. *et al.* Alcohol-Related Mortality in Patients With Psoriasis: A Population-Based Cohort Study. *JAMA Dermatol.* **153**, 1256–1262 (2017).

8. King, D. *et al.* The Outcomes of Emergency Admissions With Ulcerative Colitis Between 2007 and 2017 in England. *J. Crohns Colitis* **14**, 764–772 (2020).

9. Rye, C. *et al.* Positive Predictive Value of Diagnostic Codes for Inflammatory Bowel Disease in the Danish National Patient Registry Among Individuals 50+ Years, Using Patient Records as Reference Standard. *Clin. Epidemiol.* **13**, 335–344 (2021).

10. Abbas, N., Quraishi, M. N. & Trivedi, P. Emerging drugs for the treatment of primary sclerosing cholangitis. *Curr. Opin. Pharmacol.* **62**, 23–35 (2021).

11. Keogh, R. H., Tanner, K., Simmonds, N. J. & Bilton, D. The changing demography of the cystic fibrosis population: forecasting future numbers of adults in the UK. *Sci. Rep.* **10**, 10660 (2020).

12. Office for National Statistics. Estimates of the population for the UK, England and Wales, Scotland and Northern Ireland. Office for National Statistics.

13. Neale Swinnerton. monstR: Download publically available data via the ONS API.

14. Royston, P. & Parmar, M. K. B. Flexible parametric proportional-hazards and proportional-odds models for censored survival data, with application to prognostic modelling and estimation of treatment effects. *Stat. Med.* **21**, 2175–2197 (2002).

15. Jackson, C. **flexsurv** : A Platform for Parametric Survival Modeling in *R*. *J. Stat. Softw.* **70**, (2016).

16. Office for National Statistics. 2018-based population projections data files, England.

17. Office for National Statistics. *National Population Projections QMI*. https://www.ons.gov.uk/peoplepopulationandcommunity/populationandmigration/populationprojections/methodologies/nationalpopulationprojectionsqmi.
